# Huanglian Jiedu decoction remodels the periphery microenvironment to inhibit Alzheimer’s disease progression based on the “brain-gut” axis through multiple integrated omics

**DOI:** 10.1186/s13195-021-00779-7

**Published:** 2021-02-12

**Authors:** Xinru Gu, Junyi Zhou, Yanyan Zhou, Hongjie Wang, Nan Si, Wei Ren, Wei Zhao, Xiaorui Fan, Wenya Gao, Xiaolu Wei, Jian Yang, Baolin Bian, Haiyu Zhao

**Affiliations:** 1 Institute of Chinese Materia Medica, China Academy of Chinese Medical Sciences, Beijing, 100700 China; 2 Drug Research Center of Integrated Traditional Chinese and Western Medicine, Affiliated Traditional Chinese Medicine Hospital, Southwest Medical University, Luzhou, 646000 China; 3 Center for Drug Evaluation, National Medical Products Administration, Beijing, 100022 China

**Keywords:** Huanglian Jiedu decoction (HLJDD), Alzheimer’s disease (AD), Gut microflora, Neuroinflammation

## Abstract

**Background:**

In recent years, excellent results have suggested an association between the “brain-gut” axis and Alzheimer’s disease (AD) progression, yet the role of the “brain-gut” axis in AD pathogenesis still remains obscure. Herein, we provided a potential link between the central and peripheral neuroinflammatory disorders in AD progression.

**Methods:**

The Morris water maze (MWM) test, immunohistochemistry, ELISA, ProcartaPlex Multiplex immunoassay, multiple LC-MS/MS methods, and the V3-V4 regions of 16S rRNA genes were applied to explore potential biomarkers.

**Results:**

In Tg-APP/PS1 mice, gut dysbiosis and lipid metabolism were highly associated with AD-like neuroinflammation. The combination of inflammatory factors (IL-6 and INF-γ), phosphatidylcholines (PCs) and SCFA-producing bacteria were expected to be early diagnostic biomarkers for AD. Huanglian Jiedu decoction (HLJDD) suppressed gut dysbiosis and the associated Aβ accumulation, harnessed neuroinflammation and reversed cognitive impairment.

**Conclusion:**

Together, our findings highlighted the roles of neuroinflammation induced by gut dysbiosis and lipid metabolism disorder in AD progression. This integrated metabolomics approach showed its potential to understand the complex mechanisms of HLJDD in the treatment of AD.

**Supplementary Information:**

The online version contains supplementary material available at 10.1186/s13195-021-00779-7.

## Introduction

Alzheimer’s disease (AD), a chronic neurodegenerative disease, is a major cause of disability and mortality if not effectively treated. Over 40 million people worldwide are suffering from AD, especially in elderly people [1]. There are currently no preventative or disease-modifying treatments available, despite the countless investments that have been made in the war against AD. Beyond all doubt, the complexity of the aetiology of AD is the biggest challenge to overcome this problem. Now, as many as 1141 anti-AD drugs worldwide were in development. However, only 6 of them stood out and were approved by the Food and Drug Administration (FDA). Regrettably, these drugs are relatively against a single target and are mainly acetylcholinesterase inhibitors, which is not an optimal choice for AD patients with multi-pathogenesis. GV-971, a mixture of acidic linear oligosaccharides ranging from dimers to decamers (molecular weight up to ~ 1 kDa) that were approved by the FDA to carry out a phase 3 clinical for AD in 2020, suppresses gut dysbiosis and the associated phenylalanine/isoleucine accumulation, harnesses neuroinflammation and reverses the cognition impairment [2]. The success of GV-971 in anti-AD reveals that multi-target intervention breaks new ground for the drug development of AD. Traditional Chinese medicine (TCM), characterized by multiple components and multiple targets, closely coincides with this strategy.

HLJDD, a classical TCM formula used for fever relief and detoxification, consists of *Rhizoma coptidis* (Rc), *Radix scutellariae* (Rs), *Cortex phellodendri* (Cp) and *Fructus Gardeniae* (Fg) in a weight ratio of 3:2:2:3. It has been widely applied to treat cerebrovascular diseases, ischaemic stroke, and AD in many Asian countries for centuries [3–6]. In our previous studies, the chemical profile and haemodynamics of HLJDD were described in detail [7, 8]^.^ Sixty-nine compounds in HLJDD were identified, including mainly iridoids, alkaloids and flavonoids*.* Berberine (Ber), baicalin and geniposide were also representative components [7]. Recently, excellent results on the pharmacological effects of HLJDD or its components have been achieved in the treatment of AD, involving ameliorating cognitive dysfunction, lessening of the plaque burden and oxidative stress and altering lipid metabolism [9, 10]. However, the underlying mechanisms of HLJDD on the amelioration of AD are still a mystery.

Neuroinflammation is a key factor in the neurodegenerative process of AD [11]. This process involves an initial inflammatory stimulus (Aβ, pro-inflammatory cytokines, chemokines and the generation of reactive oxygen species) that triggers microglia, the resident macrophage within the central nervous system (CNS) [12–15]. In addition, monocytes recruited from the periphery can interact with microglia to influence the expression of amyloid-beta [16, 17]. So the accumulation of Aβ throughout the brain is the failure of microglia and peripherical monocytes to remove extracellular amyloid [18, 19], which reversely activates microglia and releases inflammatory cytokines, such as interleukin-1β (IL-1β), IL-6 and tumour necrosis factor (TNF-α) [20]. In addition, they secrete oxidative stress-related molecules, such as nitric oxide (NO), reactive oxygen species (ROS) and superoxide anions [21]. Glucose is normally the major energy source for the brain, but in AD, glucose metabolism dramatically decreases [15, 22–24]. However, docosahexaenoic acid (DHA) facilitates the transport of glucose into the brain by regulation of glucose transporter protein-1 (GLUT1) transporters and reducing Aβ plaque aggregation in individuals with moderate dementia and AD [25]. Furthermore, phospholipids (PLs), which act as storage depots from a complex meshwork of lipid mediators in cell membranes, are especially important in controlling neuroinflammation [26]. PLs influence the formation of Aβ peptides by affecting membrane proteins, such as APP, and β- and γ-secretase. The decrease of PCs in both the plasma and the brain are associated with impaired cognitive performance in elderly people and AD patients [27, 28].

It should be noted that neuroinflammation is not solely restricted to contributions from resident biochemical factors in the brain, as perturbations in microbial diversity are related to propagating neuroinflammation in preclinical models of AD. Gut microbiota could affect the immune system directly via activation of the vagus nerve [29, 30], which in turn triggers bidirectional communication with the CNS and links them to the cognitive and emotional centres of the brain [31–33]. The results of a recent clinical trial performed on elderly subjects with dementia support the evidence of the role of amyloid and related bacterial accumulation in the pathogenesis of cognitive damage [34]. Additionally, the dysregulation of gut microbiota is responsible for the increased permeability of intestinal barriers and the blood-brain barrier (BBB) [35]. Short-chain fatty acids (SCFAs) produced by bacterial fermentation of dietary carbohydrates can cross the BBB, modulate brain development and behaviour and regulate microglia homoeostasis [36–38]. In addition to SCFAs, gut bacteria also produce a range of substances with neuroactivity and immunomodulatory effects, including dopamine, γ-aminobutyric acid (GABA) and histamine [39–41]. Tryptophan (Trp) is metabolized in the periphery within the gut neurons and enterochromaffin cells, and centrally in the neurons of the raphe of the brain stem [42]. Dysregulation of Trp metabolism causes shifts in the balance between the Kyn and 5-HT pathways and is associated with psychiatric and neurological disorders [43].

Bile acids (BAs) are critically important for the maintenance of a healthy gut microbiota, a balance between lipid and carbohydrate metabolism, energy homeostasis and innate immunity [44, 45]. Disturbance of BAs has been demonstrated in AD progression [46, 47]. Circulating BAs were thought to provide a communication bridge between the gut and the brain, and their alteration reflects gut dysbiosis [48–50]. Bile salt hydrolase-rich (BSH) bacteria readily modify the BA profile. In turn, intestinal BAs control the growth and maintenance of commensal bacteria [51–53]. Metagenomic analyses have demonstrated that functional BSH is present in all major bacterial divisions and archaeal species in the human gut, including members of *Lactobacilli*, *Bifidobacteria*, *Clostridium* and *Bacteroides* [51, 54–56].

Plenty of evidence shown that peripheral and central inflammations play an important role in the pathogenesis of AD. The hypothesis of “brain-gut” axis will be an useful and promising exploration. In this study, we aimed to reveal the phenotypic features of APP/PS1 mice as comprehensively as possible, and provide a potential effective, safe and economical intervention strategy from TCM.

## Results

### Cognitive deficiency in Tg-APP/PS1 mice

**Fig. 1:**
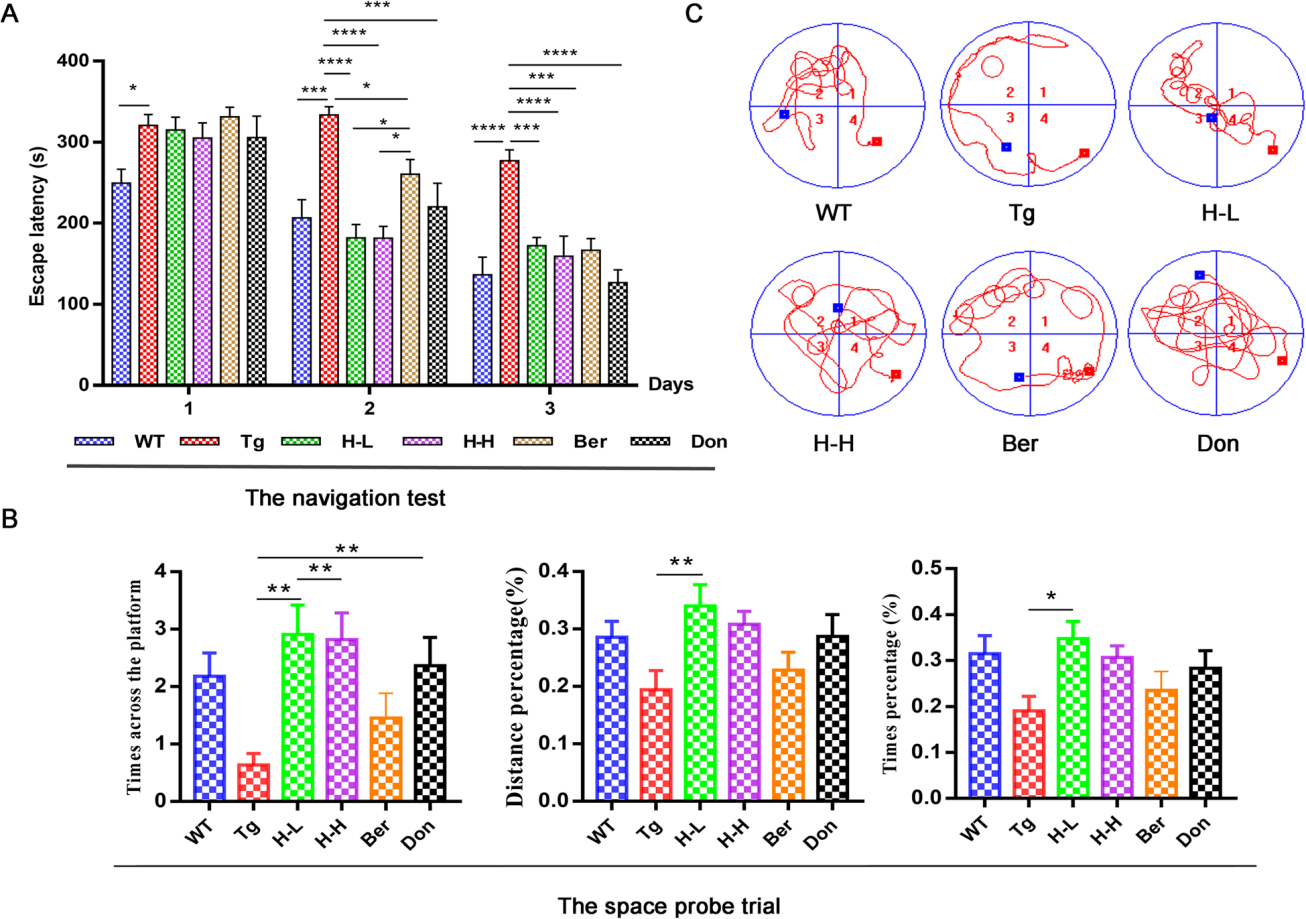
HLJDD ameliorates cognitive deficiency in APP/PS1 mice. **a** Acquisition of spatial memory was evaluated by the MWM test (n = 11). In the navigation test, mice were evaluated for the total time of four trainings spent searching for the platform location during the day. The Tg-APP/PS1 mice required a longer time than the control group to locate the platform on the training days (*P* < 0.0001). This significant difference constantly appeared from the first day. **b** In the space probe trial, the distance percentage, time percentage, and times across platform in the target quadrant were used for evaluation. All of them were reduced in Tg-APP/PS1 mice. In contrast, the chronic administration of HLJDD for 4 months, including H-L and H-H, significantly ameliorated memory and spatial learning deficits of Tg-APP/PS1 mice. **c** Real-time monitoring of the mouse motion track MWM test experiment. WT, wide mice; Tg, Tg-APP/PS1 mice; H-L, HLJDD with low dosage; H-H, HLJDD with high dosage; Ber, berberine; Don, donepezil. All data were analysed by one-way ANOVA with Dunnett-test. All the results are expressed as the mean ± SEM; *P < 0.05, **P < 0.01, ***P < 0.001, ****P < 0.0001

**Fig. 2:**
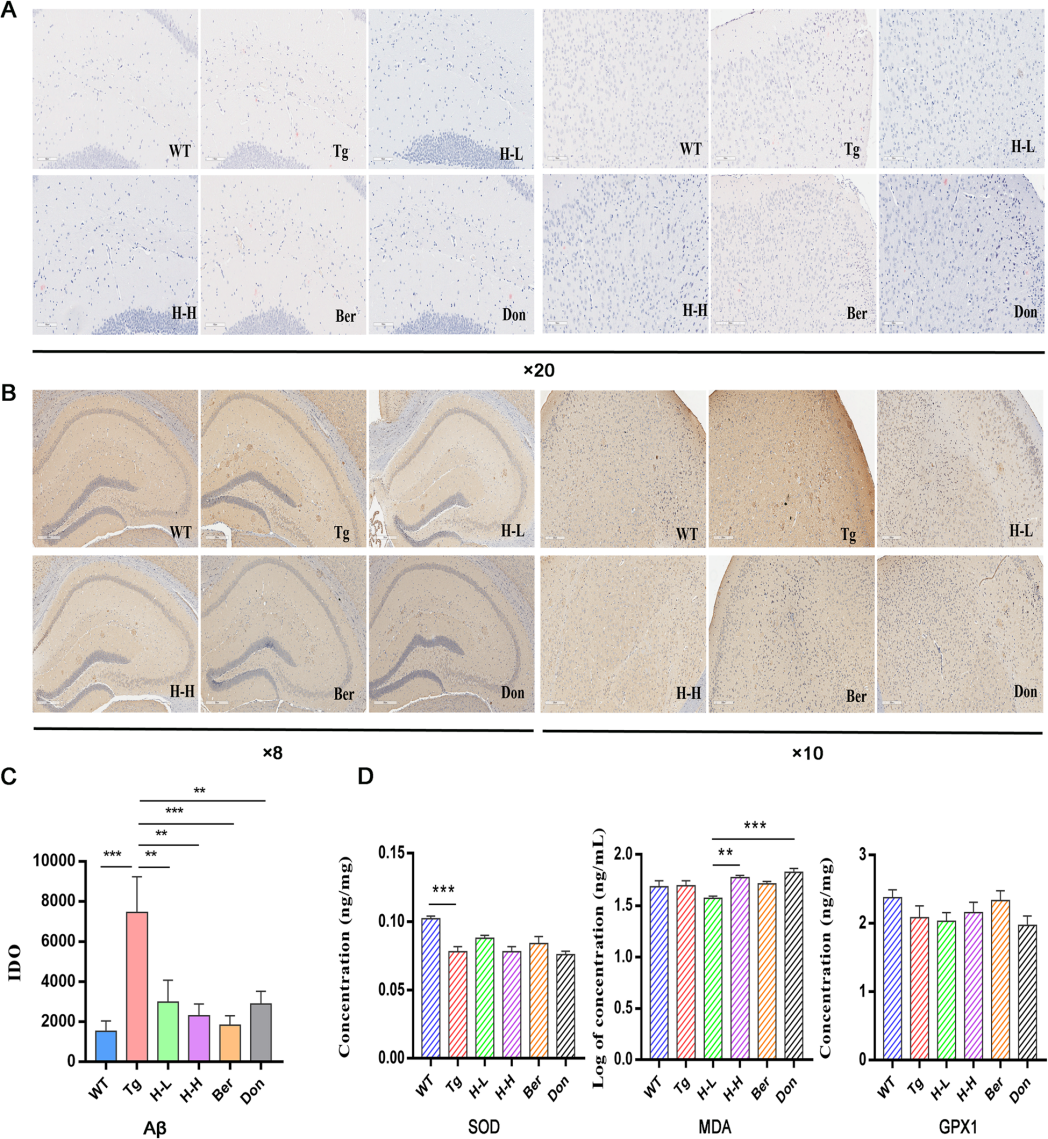
HLJDD reduced Aβ plaque pathology in Tg-APP/PS1 mice. **a** Congo red staining reveals that Tg-APP/PS1 mice harbour more Aβ plaque with higher density and larger area in the hippocampus (left) and cortex (right) than WT mice, × 20 magnification, (*n* = 3). **b** Meanwhile, the results of immunohistochemistry in the hippocampus (left, × 8 magnification) and cortex (right, × 10 magnification) of Tg-APP/PS1 mice were also positive, (*n* = 3). **c** The level of Aβ in the CNS of mice was quantified by detecting the value of Integrated option density (IOD). HLJDD significantly suppressed the accumulation of A plaques in the cortex and hippocampus. **d** The levels of brain SOD (*n* = 5), MDA (*n* = 6), and GPX1 (*n* = 6) were detected by ELISA. All data were analysed by one-way ANOVA with Dunnett-test. All the results are expressed as the mean ± SEM; *P < 0.05, **P < 0.01, ***P < 0.001, ****P < 0.0001

The cognitive functions of 8-month-old Tg-APP/PS1 (Tg) mice were significantly impaired compared to age-matched wild-type (WT) mice (Fig. 1). In the navigation test, Tg mice normally took longer time to reach the platform as compared to the WT mice over a 3-day training period (*P* < 0.0001) (Fig. 1a). In the space probe test, the times across the platform was reduced to more than half of that in WT mice (Fig. 1b). Meanwhile, the trajectory map of Tg mice was disorganized and purposeless (Fig. 1c). Aβ plaques markedly accumulated in the brain cortex and hippocampus of Tg mice compared with WT mice. Congo red staining has shown that Aβ deposition with higher density and larger area existed in the hippocampus and cortex of Tg mice than that of WT mice (Fig. 2a). Meanwhile, the results of immunohistochemistry in the hippocampus and cortex of Tg mice were also positive (Fig. 2b, c). Furthermore, the brain contents of SOD dramatically decreased in Tg mice (*P* < 0.001) (Fig. 2d). Chronic oral administration of H-L (HLJDD with low dosage: 172 mg/kg/day) and H-H (HLJDD with high dosage: 344 mg/kg/day) for 4 months significantly ameliorated the memory and spatial learning deficits of Tg mice by suppressing the accumulation of Aβ plaques in the cortex and hippocampus. In addition, H-L could increase the level of SOD in the brain of Tg mice to 1.23-fold and has a tendency to downregulate MDA. Therefore, we speculated that HLJDD has the potential to attenuate oxidative stress.

### The CNS neuroinflammation in Tg-APP/PS1 mice

#### Changes in inflammatory cytokines

**Fig. 3:**
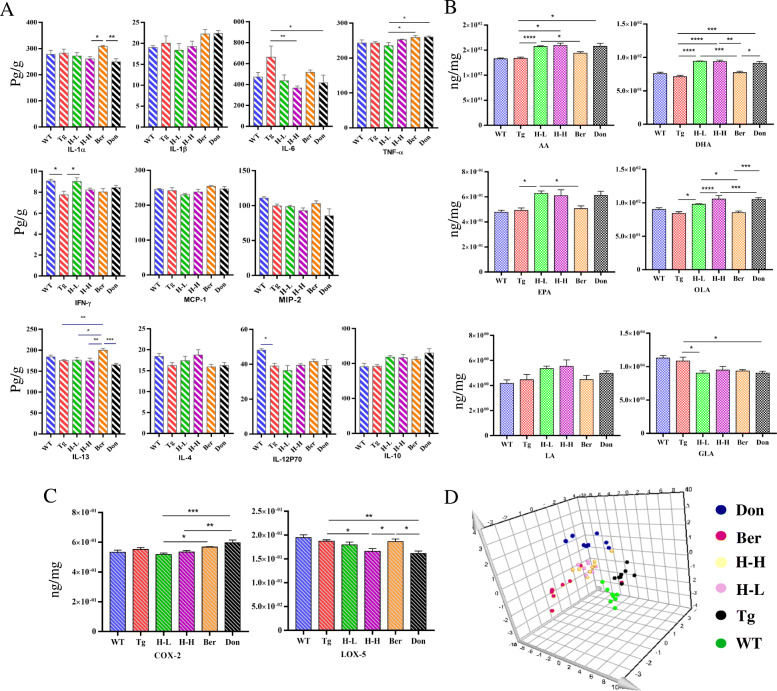
HLJDD improved the CNS inflammatory microenvironment in Tg-APP/PS1 mice. **a** Changes in pro-inflammatory and anti-inflammatory factors (*n* = 5). **b** Changes in UFAs (*n* = 6). **c** Changes in COX-2 and 5-Lox (*n* = 5). **d** PLS-DA plot. All data were analysed by one-way ANOVA with Dunnett-test. All the results are expressed as the mean ± SEM; *P < 0.05, **P < 0.01. ***P < 0.001, ****P < 0.0001

Cytokines of total brain homogenates were measured by ProcartaPlex Multiplex Immunoassay to further assess the neuroinflammatory profile (Fig. 3a). Compared with WT mice, IFN-γ and IL-12p70 were significantly decreased in Tg mice with the content of 7.75 and 38.98 pg/g. The content of IL-6 in Tg mice was about 1.4 times greater than that in WT mice. After the administration of HLJDD, the levels of IFN-γ tended to be a normal value (H-L: 9.02 pg/g; H-H: 8.22 pg/g), and the level of IL-6 observably decreased (*P* < 0.05). Furthermore, anti-inflammatory cytokine IL-4 and IL-10 both considerably increased in HLJDD mice compared with Tg mice.

#### Disorder of endogenous metabolites

Increasing evidence suggests that metabolic perturbations in various pathways mediate the occurrence of AD pathology as well as the onset of cognitive impairment in patients. UPLC-QQQ MS/MS was employed to evaluate the alteration of endogenous metabolites in Tg mice. Overall, there was no significant fluctuation in the neurotransmitters (NTs) levels in the brain of Tg mice compared with WT mice (Table 1). However, the levels of citrulline and methionine in Tg mice were higher than that in WT mice (fold: 1.66 and 1.28, respectively). HLJDD significantly increased the levels of NTs in the brain of Tg mice, including essential amino acids (phenylalanine: Phe, Trp, leucine, isoleucine and threonine), proline, choline, glutamate (Glu) and GABA, and arginine, tyrosine and asparaginate. Simultaneously, L-cysteine decreased after intervention with HLJDD. As far as PUFAs (polyunsaturated fats) were concerned (Fig. 3b), the overall pattern of results showed no significant differences in brain PUFAs of Tg mice relative to WT mice. Nevertheless, treatment with HLJDD significantly increased the brain levels of arachidonic acid (AA), DHA, eicosapentaenoic acid (EPA), linoleic acid (LA) and oleic acid (OLA) and decreased the content of γ-linolenic acid (GLA). Moreover, HLJDD treatment inhibited cyclooxygenase 2 (COX-2) and 5-lipoxygenase (5-LOX) expression in the brain of Tg mice (Fig. 3c).

**Table 1:**
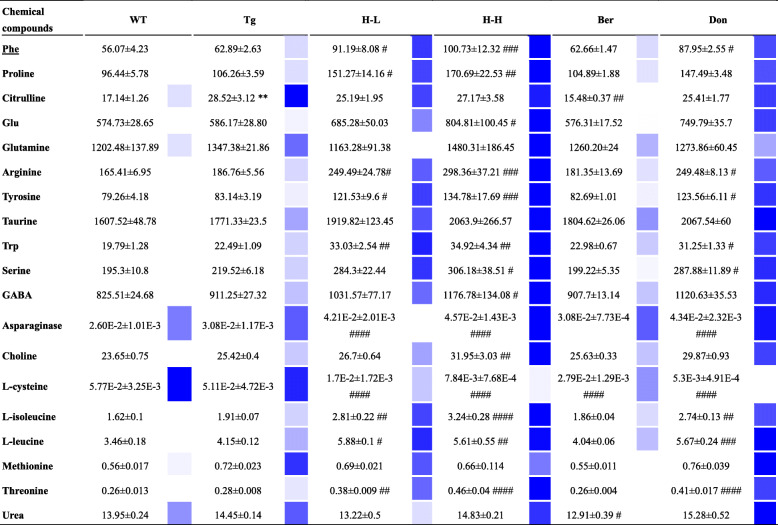
The effect of HLJDD on neurotransmitters in the CNS of Tg-APP/PS1 mice

Changes in NTs (*n* = 6–8), absolute quantification (μg/g); the remaining NTs were measured in relative quantities (A_S_/A_I_); colour coded according to the contents. The colour from light to dark represents the content from low to high. All data were analysed by one-way ANOVA with Dunnett-test. All the results are expressed as the mean ± SEM; *P < 0.05, **P < 0.01. ***P < 0.001, ****P < 0.0001 (comparing with WT mice); ^#^*P* < 0.05, ^##^*P* < 0.01. ^###^*P* < 0.001, ^####^*P* < 0.0001 (comparing with Tg mice)

In addition, dysregulation of lipid metabolism in the brains of Tg mice was confirmed by supervised OPLS-DA with the values of *R*^2^*Y* and *Q*^2^ (93% and 79%, respectively). In the PLS-DA plots (Fig. 3d), the HLJDD administration groups were relatively independent and had no intersection with Tg mice, but were closer to WT mice. In detail, 40 pathological lipid biomarkers in brain tissue were identified (Table 2). The levels of 17/21 PCs, 5/5 PEs, 3/3 glucosylceramides (GlcCers), 4/4 ceramides (Cers) and 5/7 sphingomyelins (SMs) were lower in Tg mice than in WT mice. HLJDD attenuated this lesion by increasing the contents of PCs and PEs in the brains of mice.

**Table 2:**
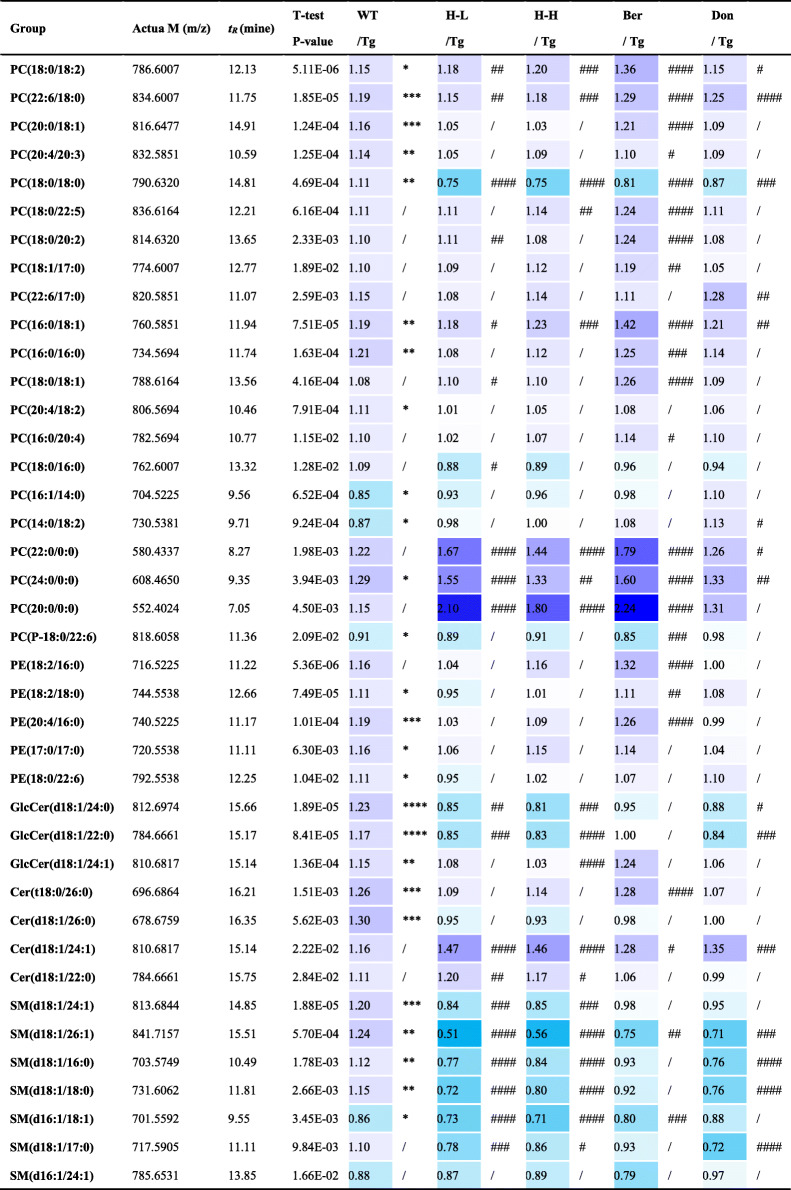
The metabolic changes of 40 potential lipids markers in the CNS of Tg mice

Colour coded according to the fold change, *n* = 8; Colour bar: ; *t*_*R*_: time retention; Actua M: actual mass; All the results are expressed as mean ± SEM; All data were analysed by one-way ANOVA with Dunnett-test; *P < 0.05, **P < 0.01, ***P < 0.001, ****P < 0.0001 (comparing with the WT group), ^#^*P* < 0.05, ^##^*P* < 0.01, ^###^*P* < 0.001, ^####^*P* < 0.0001 (comparing with the Tg group)

### The peripheral inflammation in Tg-APP/PS1 mice

#### Changes in cytokines

**Fig. 4:**
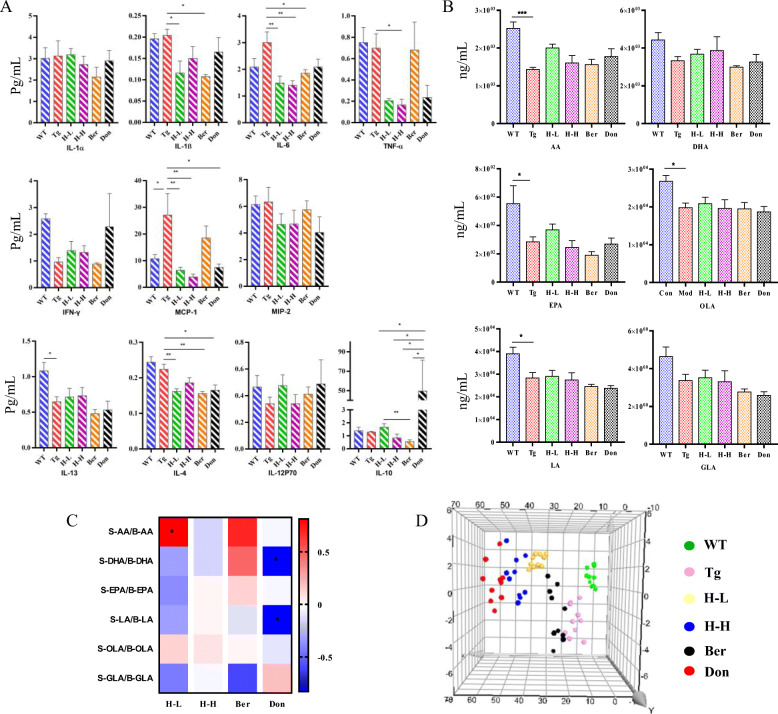
HLJDD improved the peripheral inflammatory microenvironment in Tg-APP/PS1 mice. **a** Changes in pro-inflammatory and anti-inflammatory factors (*n* = 4–5); **b** Changes in UFAs (*n* = 6). **c** Correlation analysis of the CNS UFAs and the peripheral UFAs in each drug treatment group. The colour scale illustrates the magnitude of correlation between the examined indexes on the plot. **d** PLS-DA plot. All data were analysed by one-way ANOVA with Dunnett-test. All the results are expressed as the mean ± SEM; *P < 0.05, **P < 0.01

As shown in Fig. 4a, the levels of IFN-γ, IL-13 and IL-12P70 in the serum of Tg mice (0.96, 0.65 and 0.34 pg/mL, respectively) were lower than these in WT mice (2.58, 1.08 and 0.47 pg/mL, respectively), while the contents of monocyte chemotactic protein 1 (MCP-1, 2.53-fold) and IL-6 (1.43-fold) were higher. Meanwhile, we found that the change tendency of IFN-γ, IL-6 and IL-12p70 in the periphery of Tg mice were consistent with those in the brain. Oral administration of HLJDD suppressed pro-inflammatory cytokines IL-1β, IL-6, MCP-1 and TNF-α expression in the serum of Tg mice.

#### Alteration of endogenous metabolites

An overall level of peripheral omega-6 acid and omega-9 acid decreased in Tg mice compared to WT mice (Fig. 4b), including LA (*P* < 0.05), AA (*P* < 0.001), EPA (*P* < 0.05), DHA (reduced by 25%), GLA (reduced by 28%) and OLA (*P* < 0.05). Nevertheless, the levels of serum of PUFAs showed no significant changes after HLJDD. PUFAs are required for maintaining the structure, function and vascular integrity of the brain. Non-essential fatty acids are synthesized in the brain, but essential PUFAs (e.g., AA, DHA and EPA) are largely acquired from the peripheral circulation [3]. Therefore, to investigate whether HLJDD affected central PUFAs in Tg mice by regulating peripheral PUFAs, correlation network analysis was employed based on the measured contents (Fig. 4c). Results showed that the contents of serum PUFAs (DHA, EPA, LA and GLA) were negatively correlating with those of central PUFAs, and the level of serum AA was a positive correlation with that of central AA. Dysregulation of lipid metabolism in the serum of Tg mice was also confirmed by OPLS-DA, with values of *R*^2^*x* and *Q*^2^ of 72% and 40%, respectively. The PLS-DA plot showed that HLJDD groups (including H-L and H-H) were relatively independent and had no intersection with the WT and Tg groups (Fig. 4d), which was similar to the pattern in the brain. In detail, 13 LP biomarkers in serum were screened, including 11 PCs, 1 PE and 1 GlcCers (Table 3). Almost all serum lipid biomarkers decreased in Tg mice compared with WT mice. Interestingly, all of these biomarkers were reversed by HLJDD.

**Table 3:**
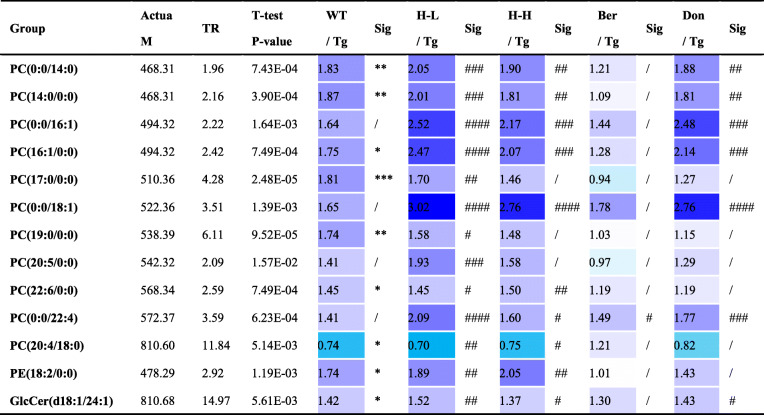
The metabolic changes of 13 potential lipids markers in the periphery of Tg mice

Colour coded according to the fold change, *n* = 10; Colour bar: ; *t*_*R*_: time retention; Actua M: actual mass; All the results are expressed as mean ± SEM; All data were analysed by one-way ANOVA with Dunnett-test; *P < 0.05, **P < 0.01, ***P < 0.001, ****P < 0.0001 (comparing with the WT group), ^#^*P* < 0.05, ^##^*P* < 0.01, ^###^*P* < 0.001, ^####^*P* < 0.0001 (comparing with the Tg group)

Additionally, we found that some significant changes occurred in peripheral BAs in Tg mice compared with WT mice (Table S1). CA was hardly detected in the periphery of WT mice, but they remained high in the serum of AD mice. An increase of tauro-conjugated BAs (taurodeoxycholic acid (TDCA), tauroursodeoxycholic acid (TUDCA), taurohyodeoxycholic acid (THDCA), tauro-α-muricholic acid (T-α-MCA)) and deoxycholic acid (DCA, almost a 30-fold increase) were observed in Tg mice compared with WT. Interestingly, HLJDD significantly decreased the levels of DCA, T-MCA and THDCA. It has been reported that the ratio between BAs reflected enzymatic activities in the liver and the gut microbiome. The cholic acid (CA) to chenodesoxycholic acid (CDCA) ratio reflected if a possible shift in BA synthesis from the primary to the alternative BA pathway has occurred in the liver. Ratios of secondary to primary BAs was used to assess the activity of intestinal microbiome enzymes which influenced the production of secondary BAs [47]. In this study, the ratio of CA:CDCA and the secondary to primary BAs were higher in Tg mice than those in WT mice, which were significantly reversed by HLJDD.

### Alteration of the gut microbiota in Tg-APP/PS1 mice

Pyrosequencing was employed to monitor the faecal microbiota composition in Tg mice. Bacterial richness and α-diversity in Tg mice showed no significant differences relative to WT, as demonstrated by the rarefaction curve, Chao index, and Shannon index (Fig. S1a-c). Moreover, HLJDD treatment did not affect the diversity of microflora in Tg mice, but Ber treatment remarkably decreased the levels of the Shannon index. According to ANOSIM analysis, the intra-group difference was less than the inter-group difference. (Fig. S1d). For PLS-DA analysis (Fig. S1e), the faecal microbiota composition of Tg mice was significantly different from that of WT mice. However, HLJDD could shift the gut microbiota composition. At the phylum level (Fig. S1f), Tg mice exhibited *Firmicutes* populations more abundantly than WT (41% and 35%, respectively), as previously reported [57, 58], while the *Bacteroidetes* population was lower (54%, 60% respectively) [59]. Treatment with HLJDD in Tg mice decreased the population of *Firmicutes* and increased the population of *Proteobacteria*. At the family level (Table 4), higher relative abundances of *Lachnospiraceae* (1.24-fold)*, Rikenellacae* (1.3-fold) and *Porphyromonadaceae* (2.24-fold) were observed in Tg mice than those in WT, while the relative abundances of *Bacteroidales_S24-7_group*, *Coriobacteriaceae* and *Alcaligenaceae* were lower (Tg: 4.13%, 0.13%, 0.16%; WT: 5.37%, 0.29%, 0.58%). Treatment with HLJDD increased the relative abundances of *Prevotellaceae*, *Lactobacillaceae*, *Peptococcaceae, Alcaligenaceae* and *Helicobacteraceae* and reduced the relative abundances of *Bacteroidales_S24-7_group*, *Lachnospiraceae* and *Porphyromonadaceae*. At the genus level (Table 5), the relative abundances of *unidentified*, *Parasutterella*, *Blautia*, *Lachnospiraceae-UCG-001* and *Ruminococcaceae-UCG-014* were lower in Tg mice (0.13%, 1.28%, 0.36% respectively) than those in WT mice (0.5%, 2.35%, 0.69% respectively), while the abundances of *Lachnospiraceae_NK4A136_ group* (1.5-fold), *Bacteroides* (5.4-fold) and *Odoribacte* (1.3-fold) were higher. However, treatment of Tg mice with HLJDD increased *Prevotellaceae_UCG_001*, *Lactobacillus*, *Helicobacter*, *Lachnospiraceae-UCG-001*, *Tyzzerella-3*, *Ruminococcaceae-UCG-014* and *Parasutterella*, and reduced the relative abundances of *g-unidentified*, *Lachnospiraceae_NK4A136_group*, *Bacteroides*, *Roseburia*, *Anaerotruncu*, *Lachnospiraceae_FCS020_group* and *Odoribacte*. In addition, LEfSe was used to identify the specific altered bacterial phenotype. A total of 44 bacteria changed significantly among the six groups, with a linear discriminant analysis (LDA) score log 10 > 3 (Fig. S2).

**Table 4:**
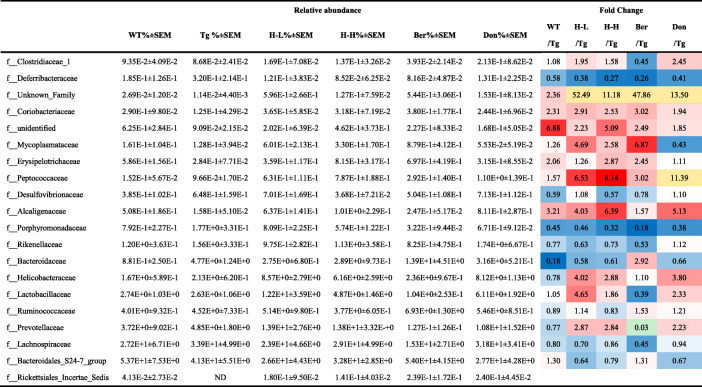
Relative abundance of the top 20 bacteria family among six groups

Colour coded according to the fold change (F). Colour bar: ; *n* = 11; Since the relative abundance of *f_Rickettsiales_Incertae_Sedis* in Tg group was zero, its fold change between groups were not calculated; ND: not detected;  : Less than 10 times;  :more than 10 times; All the results are expressed as mean ± SEM

**Table 5:**
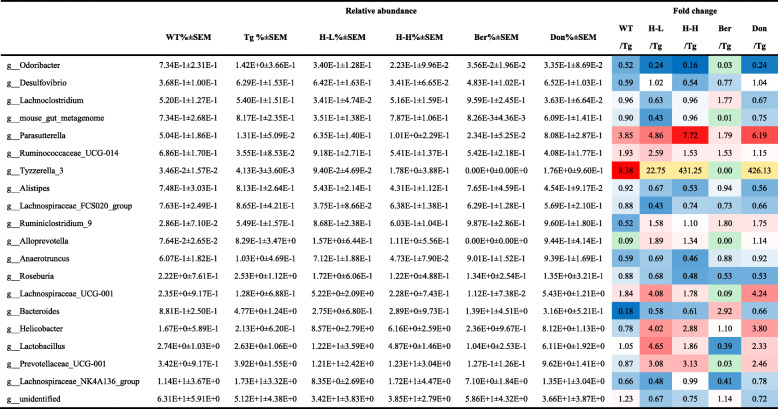
Relative abundance of the top 20 bacteria genus among six groups

Colour coded according to the fold change (F). Colour bar: ; n = 11; Since the relative abundance of *f_Rickettsiales_Incertae_Sedis* in the Tg group was zero, its fold change between groups were not calculated;  : Less than 10 times;  :more than 10 times; All the results are expressed as mean ± SEM

### Phenotypic features of Tg-APP/PS1 mice after administration of Ber

In general, Ber was not as effective as HLJDD in ameliorating cognitive deficiency in Tg mice, which was reflected in its poor performance in the MWM task and more Aβ plaques in the CNS. In terms of the therapeutic mechanism, the differences between Ber and HLJDD were mainly manifested in the regulation of neuroinflammation, lipid metabolism, and the gut microbiota. In detail, HLJDD could upregulate a variety of neurotransmitters, which was similar to Don. Ber mainly regulated citrulline, urea, and methionine. The effects of Ber on central and peripheral inflammatory cytokines were not as good as HLJDD. But Ber significantly increased the levels of IL-13 in the brain. Combining the performance of the lipid metabolic profiling of the brain with that of serum, these plot for PLS-DA presented clear separation between HLJDD (including H-L and H-H group) and Ber groups (Figs. 3d, 4d). As shown in the Venn plots (Fig. S3a), PC (O-18:0/16:0) and Cer (d18:1/24:1) were the characteristic markers of the HLJDD groups in the CNS. PC (18:1/17:0), PC (16:0/18:1), PE (22:6/0:0), PE (18:0/18:1) and PE (0:0/18:1) were the characteristic markers of the HLJDD groups in the periphery. PC (18:0/18:2) was the characteristic marker of the Ber group (Fig. S3b). Moreover, we found that Ber was similar to HLJDD in regulating the CNS pathological lipids, but its effect on peripheral pathological lipids was not as good as HLJDD. Compared with HLJDD, Ber had a weaker regulatory effect on DHA, EPA and OLA. In addition, the mechanism of Ber in regulating gut microflora was different from that of HLJDD (Tables 4, 5). After Ber administration, the proportion of special enterobacterium (*Prevotellaceae*, *Odoribacter*, *Tyzzerella_3*, *Alloprevotella*, *Lachnospiraceae_UCG-001* and *Prevotellaceae_UCG-001*) in Tg-APP/PS1 mice decreased sharply. Moreover, it was found that Ber had the opposite regulatory effect compared with HLJDD on *Prevotellaceae*, *Clostridiaceae*, *Bacteroidaceae*, *Lactobacillaceae*, *Bacteroidales_S24-7_group*, *Lachnoclostridium*, *Tyzzerella_3*, *Alloprevotella*, *Lachnospiraceae_UCG-001*, *Bacteroides*, *Lactobacillus* and *Prevotellaceae_UCG-001*.

## Discussion

**Fig. 5:**
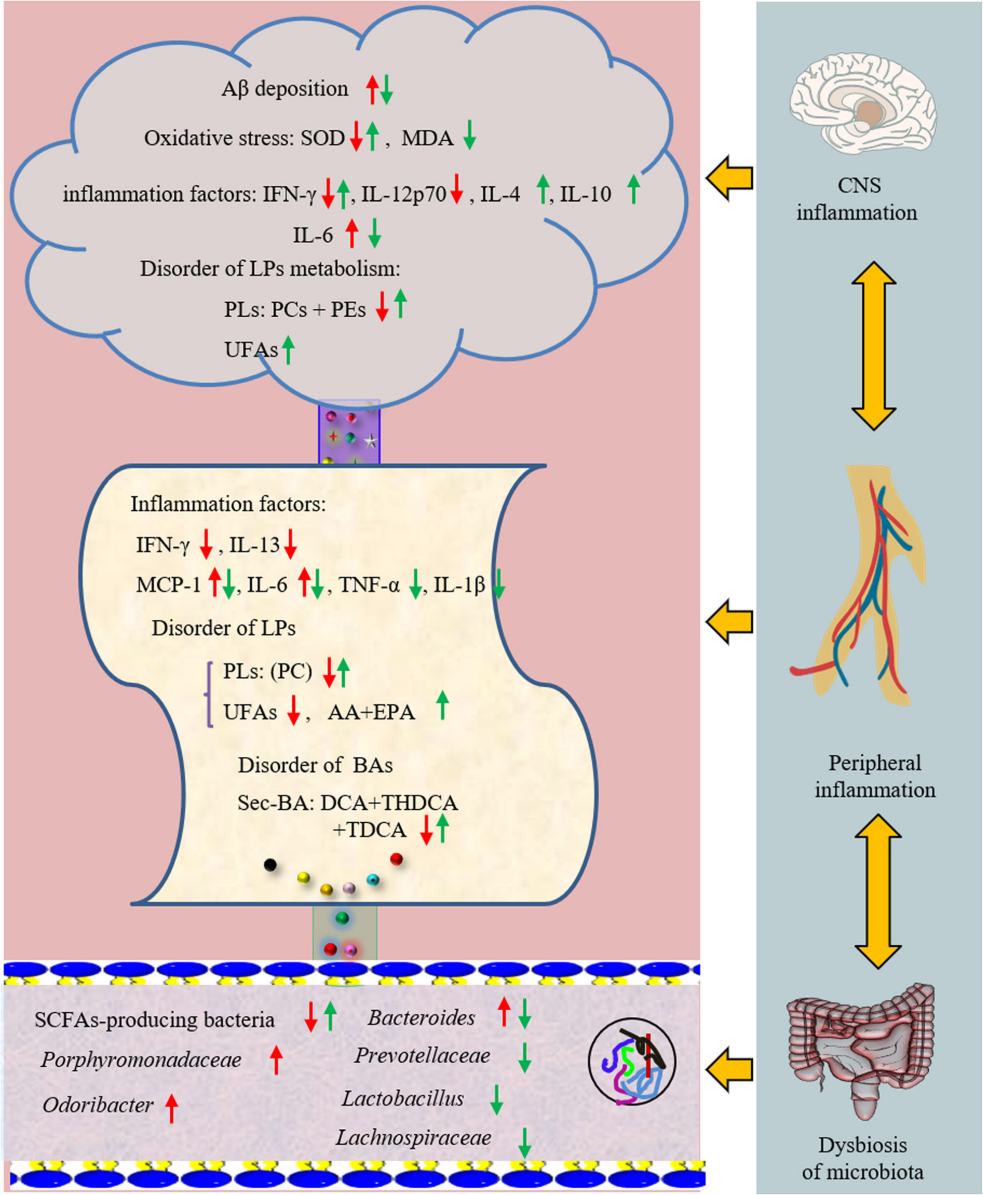
Schematic diagram of gut-brain axis in AD progression and the intervention strategy. During AD progression, peripheral inflammation was highly correlated with neuroinflammation suggests that altered systemic inflammatory markers reflect neurodegenerative disease. HLJDD attenuated brain damage and memory deficits in Tg-APP/PS1 mice by coordinating CNS inflammation and peripheral inflammation. Red arrow: represent a major change in Tg mice. Green arrow: represent a major change that occurs after the HLJDD group

In the present study, the central neuroinflammation was closely related to the onset of AD. The disorders in LPs metabolism and intestinal flora were the potential drivers (Fig. 5). 16S rRNA gene sequencing of the gut microbiome and integrated metabolomics were adopted to monitor the phenotypic features of APP/PS1 mice. The disorder of both biochemical factors in the brain and the microbial diversity in intestine contributed to the neuroinflammation. HLJDD could reverse them and then improve the cognitive impairment in Tg mice.

The elevation of Aβ level induced the overexpression of pro-inflammatory cytokines and chemokines to robust inflammatory response [20]. The more severe inflammatory degree was found in the brain of Tg mice than that in WT mice, followed with Aβ plaques accumulation and abnormal expression of inflammatory cytokines, such as IFN-γ, IL-12p70 and IL-6. Meanwhile, the elevated level of Aβ_42_ was closely associated with the increased level of oxidative stress [60], which was mainly reflected in the decrease of SOD and the increase of methionine in Tg-APP/PS1 mice. Methionine could increase the levels of Aβ and nitro-tyrosinated protein, which further induced neuroinflammation [61, 62]. In addition, the evidence showed that inflammation in Tg mice was not only confined to the CNS but also spread to the periphery. Compared with WT mice, both IFN-γ and IL-6 in the CNS and periphery of Tg mice showed significant changes. Upregulation of IL-6 was predictive of progression to AD, which was verified in various AD models and patients with varying degrees of AD [9, 63]. Study reported that INF-γ has dual roles in Alzheimer’s disease [64]. Browne et al. reported that the release of INF-γ from infiltrating Th1 cells plays a diabolical role in AD pathogenesis. There was still reports that INF-γ at the brain’s choroid plexus were reduced in brain ageing [65, 66] and under neurodegenerative condition [67]. The availability of INF-γ at the brain of AD mice might be affected by systemic immune suppression. Meanwhile, the higher level of MCP-1 in the serum was found in Tg mice compared with WT mice, which appeared to be associated with greater severity and a faster cognitive decline [68, 69].

Many Aβ-produced proteins have been found in lipid rafts such as Aβ protein precursor (βAPP), β-secretase, γ- secretase and neprilysin [70]. In addition, Aβ directly disrupt bilayer integrity by interacting with PLs [71]. Data from targeted lipidomics showed that Tg mice were characterized by a decrease in PCs, PEs and SMs of the CNS or periphery. Previous evidence has shown that a reduced concentration of PCs in the CNS and peripheral system was associated with impaired cognitive performance in older individuals and AD patients [27, 28, 72]. Among them, PC (16:0/16:0) was predictive of progression to AD dementia in individuals with mild cognitive impairment (MCI) [73, 74]. Oxidative and lipid peroxidation were early events in AD [75]. In this study, the results of PEs, PCs and SOD were in accordance with the previous paper, which suggested the implication of oxidative stress in the progressive degradation of brain PLs in AD. PCs and PEs were rich in readily oxidizable AAs and DHAs [76, 77]. We speculated that the metabolic disorder of PC and PE induced the abnormal expression of Aβ by breaking down the lipid rafts homeostasis, which deteriorated neuroinflammation.

PUFAs act as precursors for biosynthesis of the lipid mediators, which are actively involved in the inflammatory response. We found that the levels of brain PUFAs had no significant difference in Tg mice compared to WT mice. Others have shown that the brain PUFAs differ very little in AD compared to healthy people [78–80]. However, the contents of PUFAs and OLA in the periphery of Tg mice decreased significantly compared with those of WT mice. A higher level of AA is strongly associated with AD by yielding some inflammatory mediators [81, 82]. Conversely, some study still believes that a lower level of AA is associated with cognitive decline [83, 84]. In this study, the level of AA in the periphery of Tg mice decreased compared with WT mice, which was attributed to the decline of upstream molecules, including OLA, LA and GLA. LA, GLA and OLA participate in numerous cellular functions by affecting membrane fluidity, membrane enzymatic activities and eicosanoid synthesis [84].

The present study in Tg mice indicated that alterations in the gut microbiome contributed to neuroinflammation. Study reported that the circulating omega-3 fatty acid (DHA, EPA) can influence the composition of the host gut flora, especially SCFA-producing bacteria [85]. SCFA-producing bacteria decreased in Tg mice compared with the WT mice, including *Parasutterella* and *Blautia*. SCFAs can interact with nerve cells by stimulating the sympathetic and autonomic nervous system [86, 87]. SCFAs even cross the BBB and regulate microglial homeostasis. A decrease of SCFA-producing bacteria aggravated cognitive decline. *Porphyromonadaceae* is highly associated with inflammatory diseases [88, 89], and even induces cognitive decline and anxiety-like behaviour [90]. Increases in bacterial taxa from this family have also been observed in faecal samples from individuals with major depressive disorders, especially *Odoribacter* [91]. An increase in intestinal *Odoribacter* was certified in a variety of AD models [58, 90, 92]. Likewise, we observed that the proportion of *Porphyromonadaceae* and *Odoribacter* increased in Tg mice compared with WT mice, which expected to be a potential biomarker to predict the occurrence of AD.

Circulating BAs provide an important mechanism for communication between the gut and the brain [48, 93, 94]. Alterations in the gut microbiome significantly affect BAs transformation through various microbial enzymes such as bile salt hydrolase (BSHs) and hydroxysteroid dehydrogenases [95]. Compared with WT mice, an increase of tauro-conjugated BAs was observed in Tg-APP/PS1 mice, probably a result of lower BSHs activity from Lactobacillus. Indeed, Lactobacillus have BSHs activity for catalyzing deconjugation of tauro-conjugated BAs [96]. Increased amounts of secondary BAs in the blood may enter the brain through the permeability of the BBB and affect brain physiology and metabolism [97]. DCA represent aetiological agents and mediators of the pathogenic process [98, 99]. A higher level of DCA was significantly associated with worse cognitive performance [100]. The decline of CA level is thought to be one of the causes of cognitive impairment in AD patients [47]. The decline of CA in Tg-APP/PS1 mice may be caused by species difference [47, 100]. In addition, the ratios of secondary to primary BAs increased in Tg mice relative to WT mice. This indicated that the enzymatic steps in the conversion of primary to secondary BAs in the gut might contribute to disease.

The present study demonstrated that HLJDD with strict quality control could reverse cognitive impairment in Tg mice. It is worth mentioning that HLJDD decreased the expression of IL-6 in both of brain and serum of Tg mice. Upregulation of IL-6 forced microglia polarization to pro-inflammatory and neurodegeneration [101]. In addition, the regulation of HLJDD on central NTs was similar to that of Don. The previous literature reported that HLJDD ameliorates the cognitive dysfunction by regulating LP metabolism [9]. The present study indicated that HLJDD could relieve the neuroinflammation by increasing the levels of PCs, PEs, DHA and EPA. In the correlation network analysis, GLA, OLA, LA, EPA and DHA were negatively correlated at the central and peripheral levels after administration of HLJDD. However, the contents of AA in periphery showed a positive association with that in the CNS. These results suggested that HLJDD regulated the performance of central PUFAs by alleviating peripheral PUFAs, since these essential PUFAs in the brain are primarily derived from the peripheral circulation [102]. In addition, we observed that HLJDD effectively reshaped the gut microbiota structure in Tg mice. HLJDD enriched the gut microbiota SCFA-producing bacteria population. The circulating DHA was positively correlated with SCFA-producing bacteria. Therefore, we speculated that HLJDD may alter the microbiota composition by affecting the levels of DHA in Tg mice. Meanwhile, HLJDD could increase the populations of *Prevotellaceae* and its genus, including *Prevotellaceae_UCG-001*, *Prevotellaceae_Ga6A1_group* and *Parasutterella*. These bacteria were reported to be reduced in PD patients and Tg mice [103, 104]. *Prevotella* is known to breakdown complex carbohydrates, providing SCFAs as well as thiamine and folate as by products that promote a healthy intestinal environment. The decrease of *Prevotella* may reduce the levels of these important micronutrients.

The previous study demonstrated that HLJDD restored the dysregulated microbiota structure and function by increasing SCFA-producing bacteria in T2DM rats [105]. *Lachnospiraceae* can induce cognitive defect in AD patients [106] by intervening in the mucosal integrity of the host, BA, glucose and lipid metabolism [107]. In addition, HLJDD could remodel serum BAs homeostasis in Tg mice by decreasing the populations of *Lachnospiraceae* and its followed genera and increasing the populations of *Bacteroides* and *Lactobacillus.* Bacteroides and Lactobacillus were reported to be actively involved in BAs metabolism, including deconjugation, the hydroxyl groups at C3, C7 and C12, and desulfatation [108]*.* The ratios of secondary to primary BAs changed in Tg mice, which proved that HLJDD affected BAs metabolism by alternating microbiota structure. The ratio of secondary BAs to primary BAs is used to assess differences in the activity of intestinal microbiome enzymes that can induce BAs changes [47].

### Limitations

This study had some limitations. Only MWM test was performed to assess short term memory which is impaired in AD. Actually, various behavioural assessments were indeed the best strategy for the comprehensive evaluation of memory. In addition, although we have revealed existing communication disorders of the “brain-gut” axis in the pathogenesis of AD, further studies to identify the key bacterial strains and functional enzymes that may account for metabolites and neuroinflammation changes will be critical. Further research on these key issues may help gain a better understanding of the pathogenesis of AD and perfectly elucidate the potential mechanism of HLJDD in the treatment of AD.

## Conclusion

In the present study, neuroinflammation was closely related to the onset of AD, and disorders in LP metabolism and intestinal flora, which were the potential drivers. Peripheral inflammation displayed the crosstalk with CNS inflammation and cognitive impairment. The combination of inflammatory factors (IL-6 and INF-γ), PCs and SCFA-producing bacteria was a promising early diagnostic biomarker for AD, which worths further validation in numerous experiments. HLJDD suppressed gut dysbiosis and the associated Aβ accumulation, harnessed neuroinflammation and reversed cognitive impairment. More importantly, the identified anti-inflammatory actions of HLJDD will open a new therapeutic avenue for AD treatment through an intervention pattern in the “brain-gut” axis and guide the future development of effective therapies by the central-peripheral contrast study. Further research should focus on the causal relationship between the peripheral and central in AD models and patients, which may help gain a better understanding of the pathogenesis of AD and the potential mechanism of HLJDD in the treatment of AD.

## Supplementary Information

**Additional file 1.** Materials and Methods.

**Additional file 2: Table S1.** Levels and ratios of Bile acids measured in the Tg mice (*n* = 5). **Table S2.** Peak areas of three main compounds in HLJDD. **Table S3.** The selected detecting ions, Declustering potential (DP), and collision energy (CE) of neurotransmitters. **Table S4.** The regression equations and linear range of NTs. **Table S5.** The selected detecting ions, declustering potential (DP), and collision energy (CE) of UFAs. **Table S6.** The regression equations and linear range of UFAs. **Table S7.** The selected detecting ions, declustering potential (DP), and collision energy (CE) of BAs. **Table S8.** The regression equations and linear range of BAs. **Fig. S1.** HLJDD altered the overall gut microbiota structure in Tg mice (*n* = 11). (a) Rarefaction analyses; (b) Chao1 index (data expressed as mean ± SEM); (c) Shannon index (data expressed as mean ± SEM); (d) ANOSIM analysis, R-value > 0 means the intra group difference was less than the inter group difference. (e) PLS-DA analyses; (f) Bacterial taxonomic profiling in the phylum level of gut microbiota. #*P* < 0.05 (comparing with the Mod group). **Fig. S2.** LEfSe rank plots of differentially abundant microbial clades in gut microbiome (n = 11). LDA scores (log 10) for differentially abundant microbial clades in stool among six groups and the threshold on the logarithmic LDA score for discriminative feature is > 3.0. **Fig. S3.** Venn diagram of the brain (a) and serum (b) showed the shared and unique correlated lipid compounds, in which each ellipse represented the potential lipid markers based on comparisons of the Tg group vs. drug treatment groups. **Fig. S4.** Profile of HPLC-UV chromatograms of mixed standard (a), HLJDD-1st (b) and HLJDD-7th (c) . peaks: 1. Geniposide; 2. Beiberine; 3. Baicalin. The chemical pattern of HLJDD aqueous did not change significantly after 7 days of preparation.

## Data Availability

The datasets used and/or analysed during the current study are available from the corresponding author on reasonable request.

## Abbreviations

5-HT: Serotonin
5-LOX: 5-Lipoxygenase
AA: Arachidonic acid
AD: Alzheimer’s disease
ApoE: Apolipoprotein E
APP: Amyloid precursor protein
Aβ: Amyloid β-protein
BA: Bile acid
BBB: Blood-brain barrier
BSH: Bile salt hydrolase-rich
Ber: Berberine
CA: Cholic acid
CDCA: Chenodesoxycholic acid
Cer: Ceramide
CNS: Central nervous system
COX-2: Cyclooxygenase 2
Cp: *Cortex phellodendri*
DCA: Deoxycholic acid
DHA: Docosahexaenoic acid
Don: Donepezil
ELISA: Enzyme-linked immunosorbent assay
EPA: Eicosapentaenoic acid
FDA: Food and Drug Administration
Fg: *Fructus Gardeniae*
GABA: γ-Aminobutyric acid
GLA: γ-Linolenic acid
GlcCers: Glucosylceramides
Glu: Glutamate
GLUT1: Glucose transporter protein-1
GPX-1: Glutathione peroxidase 1
HETEs: Hydroxyeicosatetraenoic acids
HLJDD: Huanglian Jiedu decoction
H-L: HLJDD with low dosage
H-H: HLJDD with high dosage
Rs: *Radix scutellariae*
Rc: *Rhizoma coptidis*
IBD: Inflammatory bowel disease
IFN-γ: Interferon-γ
IL-1β: Interleukin-1β
IS: Internal standard
Kyn: Kynurenine
LA: Linoleic acid
LCA: Lithocholic acid
LDA: Linear discriminant analysis
LTs: Leukotrienes
MCI: Mild cognitive impairment
MCP-1: Monocyte chemotactic protein 1
MDA: Malonic acid
MIP-2: Murine microphage inflammatory protein-2
MWM: Morris water maze
NO: Nitric oxide
OPLS-DA: Orthogonal partial least squares discriminant analysis
PC: Phosphatidylcholine
PE: Phosphatidyl ethanolamine
PEMT: Phosphatidylethanolamine N-methyltransferase
PG: Prostaglandin
Phe: Phenylalanine
PLA2: Phospholipase A2
PLs: Phospholipids
PLS-DA: Partial least squares discriminant analysis
ROS: Reactive oxygen species
SCFAs: Short-chain fatty acids
SMs: Sphingomyelins
SOD: Superoxide dismutase
T_2_DM: Type 2 diabetes mellitus
TCA: Taurocholate
TCDCA: Taurochenodeoxycholate acid
TCM: Traditional Chinese medicine
TDCA: Taurodeoxycholic acid
Tg mice: APP/PS1 transgenic mice
THDCA: Taurohyodeoxycholic acid
TNF-α: Tumour necrosis factor α
Trp: Tryptophan
TUDCA: Tauroursodeoxycholic acid
T-α-MCA: Tauro-α-muricholic acid
UDCA: Ursodeoxycholic acid
PUFAs: Polyunsaturated fatty acids
WT: Wild mice
