# Supplementary material for: Huanglian Jiedu decoction remodels the periphery microenvironment to inhibit Alzheimer’s disease progression based on the “brain-gut” axis through multiple integrated omics
Source: Alzheimers Res Ther. 2021 Feb 12;13:44. doi: 10.1186/s13195-021-00779-7 (PMC7881564; doi:10.1186/s13195-021-00779-7)
Supplement: Supplementary file 1 — Additional file 1. Materials and Methods. [file 13195_2021_779_MOESM1_ESM.docx]

**Supplementary Materials and Methods**

**Chemicals and Reagents**

Proline, GABA, tyrosine, citrulline, glutamate (Glu), and arginine were purchased from Sigma (St, Louis, MO, USA). Phenylalanine (Phe), tryptophan (Trp), L-isoleucine, L-leucine, taurine, γ-linolenic acid (GLA) and methionine were provided by J&K Scientific (Beijing, China). Glutamine and serine were purchased from National Institutes for Food and Drug Control (Beijing, China). L-cysteine, urea, choline, asparaginase, threonine, eicosapentaenoic acid (EPA), DHA, oleic acid (OLA), linoleic acid (LA), [deoxycholic](javascript:;) [acid](javascript:;) (DCA), ursodeoxycholic acid (UDCA) and [lithocholic](javascript:;) [acid](javascript:;) (LCA) were obtained from Aladdin (Shanghai, China). Arachidonic acid (AA) was provided by the Tokyo Chemical Industry (Tokyo, Japanese). Cholic acid (CA), taurohyodeoxycholic acid (THDCA), taurodeoxycholic acid (TDCA), [chenodesoxycholic](javascript:;) [acid](javascript:;) (CDCA), and tauro-α-muricholic acid (T-α-MCA) were obtained from Trc Co. Inc. (Toronto, Canada). Tauroursodeoxycholic acid (TUDCA), taurochenodeoxycholic acid (TCDCA), and taurocholate (TCA) were purchased from Beijing Sciherbs Scitech Co. Ltd. (Beijing, China). HPLC grade methanol and acetonitrile for qualitative analysis and extraction were obtained from Honeywell Burdick and Jackson (Swedesboro, NJ, USA). HPLC grade formic acid was provided by Thermo Fisher Scientific (Bremen, Germany). Ultra-pure water was purified with a Millipore system (Millipore, Billerica, MA, USA). Other chemicals and solvents were of analytical grade.

**Drug treatment and animals**

Four samples of dried and crushed plant material were homogenized at a ratio of 3:2:2:3 (Rc: Rs:Cp:Fg) and decocted twice with boiling water (1:10, w/v) for 2 h. All herbs were authenticated by professor He Xi-Rong [[1](#_ENREF_1)], the Institute of Chinese Materia Medica, China Academy of Chinese Medical Sciences. The aqueous extract was concentrated to a constant weight on a rotary vacuum evaporator at 80 °C and crushed into powder before the experiment. The HLJDD extract powder, Ber, and Don were individually dissolved in  [distilled](javascript:;) [water](javascript:;) vi*a* ultrasonication at a certain concentration. All the aqueous were prepared once a week and stored at 4 ℃. Berberine hydrochloride tablets were provided by Chengdu Jinhua Pharmaceutical Co., Ltd. (Chengdu, China). Don was obtained from Eisai China Inc. (Suzhou, China).

To investigate the stability of HLJDD aqueous, HPLC analysis was performed on HLJDD aqueous solution on day 1 and day 7. 5 mL of HLJDD aqueous was taken for ultrasonically extracting with 15 mL methanol at ambient temperature for 30 minutes, and then filtered through a 0.22 μm membrane filter prior to injection to the HPLC system. The mixed standard solution (including berberine, baicalin, and geniposide) was prepared in methanol. The HPLC analyses were performed on Shimadzu LC 20AT (Shimadzu, Kyoto, Japan). Chromatographic separation was performed on an Eclipse XDB-C18 column (4.6×250 mm, 5 μm) with a solvent flow rate of 1 mL/min at a temperature of 30 ℃. The mobile phase was composed of water (A: containing o.1% formic acid) and methanol (B). The solvent gradient adopted was as follows: 0-5 min, 10-30% B; 5-20 min, 30-40% B; 40-50 min, 50-60% B; 50-60 min, 60-100% B. The injection volume was 10 μL. The samples was detected at 254 nm. Profile of HPLC-UV chromatograms were showed in Fig. S4. Peak areas of three main compounds in HLJDD was listed in Table S2.

Tg-APP/PS1 mice (Male, 4 months old, C57/BL6 background) and C57BL6J mice (Male, 4 months old) with body weights ranging from 28-32 g were purchased from Beijing HFK Bioscience Co., Ltd. (Beijing, China). All experiments and animal care in this study were conducted in accordance with the National Institutes of Health Guide for the Care and Use of Laboratory Animals (NIH Publications No. 8023, revised 1978) and the Provision and General Recommendation of Chinese Experimental Animals Administration Legislation. The animals were raised in single cage and kept under a consistent temperature (24 °C) with a 12 h light/dark cycle, fed standard food pellets and had access to sterile water. After acclimatization for 4 days, all Tg-APP/PS1 mice were randomly divided into 5 groups with 11 mice and were given the drugs orally by gavage every day: Tg-APP/PS1 group (Tg: saline), HLJDD-low group (H-L: 172 mg/kg/d), HLJDD-high group (H-H: 344 mg/kg/d), berberine group (Ber, 100 mg/kg/d), and donepezil (Don, 2 mg/kg/d). The remaining wild-type mice served as the control group (WT, wild mice treated with saline by gavage, n=11)

**The MWM test**

The MWM test was performed to detect spatial memory as previously described with a slight modification [[2](#_ENREF_2)]. Mice participated in a navigation test for three consecutive days. Four sequential training trials began by placing the animals facing the wall of the pool but changing the drop position for each trial. If a mouse failed to find the platform within 90 s, it was guided to the platform and remained there for 20 s. The total time to find the platform was defined as the escape latency. On the fourth day, the mice were allowed to swim freely for 60 s in different directions without a platform. Finally, the distance, time, and platform-crossing times in the target quadrant were monitored to evaluate the capability of the spatial probe.

**Sample collection**

After the MWM test, all mice rested for four days under normal conditions and then were sacrificed with choral hydrate. After anaesthesia, the whole blood was collected from the heart and centrifuged at 5 000 g for 10 min at 4 °C to obtain serum. Then, the brains were separated in sequence and washed in saline until they were colourless. Finally, 2 to 3 faces were obtained from the large intestine and placed into liquid nitrogen immediately. All the samples collected were stored at -80 °C until analysis.

**Immunohistochemistry and image analysis**

Three mice in each group were randomly singled out for Congo red staining and immunohistochemistry. Mice were anaesthetized with 2% chloral hydrate (0.2 mL/10 g) and the brain was removed from the skull and post-fixed in 4% paraformaldehyde. The slices were blocked with 3% BSA in PBS for 30 minutes at room temperature followed by an overnight incubation at 4℃ with goat anti-rabbit IgG (G23303) against Aβ (GB 13414-1) diluted in PBS. After primary antibody incubation, the slices were washed with PBS and then incubated with Aβ (GB 13414-1) secondary antibodies for 50 minutes at 37℃ in the dark, and then rinsed three times with PBS. Nuclear staining was performed using hematoxylin for 3 minutes followed by exhaustive washing in distilled water. And then the slices were subsequently examined using a microscope.

**ELISA**

Cell lysis buffer (EPX-99999-000) was added into the brain homogenate weighed precisely to further obtain the homogenate (20 mg/mL), followed by vortexing and centrifugation at 12 000 rpm/min or 15 min. Superoxide dismutase (SOD: SES134Mu), malonic acid (MDA: CEA597Ge), glutathione peroxidase 1 (GPX-1: SEA29Mu), cyclooxygenase 2 (COX-2: ab210574), and 5-lipoxygenase (5-LOX: SEB335Mu) measurements were performed according to the manufacturer’s instructions. All samples were tested twice in parallel.

**Measurement of cytokines with the ProcartaPlex Multiplex Immunoassay**

Cell lysis buffer (EPX-99999-000) was added into brain homogenate weighted precisely to further obtain the homogenate (5 mg/mL), followed by vortex and centrifugation at 12.000 rpm/min for 15 min. The concentrations (pg/mL) of pro-inflammatory cytokines [IL-1α, IL-1β, IL-6, monocyte chemotactic protein 1 (MCP-1), murine microphage inflammatory protein-2 (MIP-2), and TNF-α] and anti-inflammatory cytokines [interferon-γ (IFN-γ), IL-4, IL-12p70, IL-10, and IL-13] in the brain and serum samples were measured by ProcartaPlex Multiplex immunoassay with a commercial multiplex suspension array technology kit (PPX-10, eBioscience) according to the manufacturer’s instructions.

## UPLC-QQQ-MS/MS for the detection of endogenous substances

**Preparation of serum** First, 100 µL aliquots of plasma thawed at room temperature were mixed with 10 µL of ascorbic acid, 10 µL of internal standard (IS, penicillin) and 380 µL of methanol (containing 0.2% formic acid). Following vortexing and centrifugation at 12 000 rpm/min for 15 min, the aliquots were analysed.

**Preparation of brain tissue** Brain tissue was thawed at room temperature and homogenized repeatedly using a Scientz-48L homogenizer (3000 rpm, 50 Hz, -10 °C, 60 s)**.** Then, 10 µL of ascorbic acid, 10 µL of IS and 480 µL of 80% acetonitrile (containing 0.2% formic acid) were added to 50 mg of precisely weighed homogenate, and homogenized again. Following vortexing and centrifugation at 12 000 rpm/min for 15 min, the supernatants were analysed.

**UPLC-QQQ-MS/MS** LC-MS/MS analysis was performed on an Agilent 1290 HPLC system (Agilent Corporation, MA, USA) coupled with an AB Sciex QTRAP 6500 triple quadrupole tandem mass spectrometer (AB Sciex, Foster City, CA) operated with Analyst 1.6.2. Global parameters were optimized across all analytes: curtain gas: 20, IonSpray voltage (V): 5500, ion source gas 1: 55, ion source gas 2: 55, source temperature: 555 °C. Multiquant 3.0.1 (AB Sciex) was utilized for peak integration, the generation of calibration curves, and data analysis.

**Determination of neurotransmitters** The 11 neurotransmitters were dissolved in 50% methanol to obtain a stock solution. The stock solution was diluted with 50% methanol (containing 1% formic acid) to different concentrations. One hundred microlitres of standard solution was mixed with 5 µL of foetal bovine serum (FBS), 10 µL of ascorbic acid, 10 µL of IS (evodiamine dissolved in 50% methanol), and acetonitrile (containing 0.2% formic acid). Then, the samples were vortexed and centrifuged at 12 000 rpm/min for 15 min. Brain sample preparation was described as in ***Preparation of brain tissue.*** Separation was achieved using a Waters ACQUITY UPLC BEH amide (2.1×100 mm, 1.7 μm) column maintained at 30 °C. The mobile phase consisted of water containing 20 mM [ammonium](javascript:;) [acetate](javascript:;) (A), and acetonitrile (B) and was used at a flow rate of 0.3 mL/min. The gradient program was as follows: 95%-70% B from 0 to 20 min, 70%-50% B from 20 to 21 min, and 50% B from 21 to 24 min. The injection volume was 3 μL. The optimized mass transition ion pairs (*m/z*), CE, and DE values for the PUFAs are shown in Table S3**.** All the calibration curves indicated good linearity, with correlation coefficients (r) ranging from 0.926 to 0.998 (Table S4).

**Measurement of UFAs** The UFA standards were dissolved in methanol to obtain a stock solution. One hundred microlitres of the stock solution was successively mixed with 5 µL of foetal bovine serum (FSA), 10 µL of ascorbic acid (dissolved in physiological saline, w/v: 1 g/100 mL), 10 µL of IS (penicillin for serum and evodiamine for brain tissue), and organic regent (375 µL of methanol for serum and 375 µL of 80% acetonitrile for brain tissue; both containing 0.2% formic acid). Then, the samples were vortexed and centrifuged at 12 000 rpm/min for 15 min. The preparation of serum and brain samples was described as in ***Preparation of serum*** and ***Preparation of brain tissue****,* respectively. The separation was achieved at 30 °C using a Thermo Hypersil GOLD column (2.1×50 mm, 1.9 μm). The mobile phase consisted of water containing 0.1% formic acid (A) and methanol (B) at a flow rate of 0.4 mL**/**min. The gradient program used was as follows: 10% B from 0 to 1 min, 40% B from 1 to 3 min, and 60%–100% B from 3 to 7 min. The injection volume was 3 μL. The optimized mass transition ion pairs (*m/z*), CE, and DE values for the PUFAs are shown in Table S5**.** All the calibration curves indicated good linearity with good correlation coefficients (r) (Table S6).

**Determination of BAs** The 7 BA standards were dissolved in methanol to obtain a stock solution. One hundred microlitres of the stock solution was successively mixed with 5 µL of foetal bovine sera (FSA), 10 µL of ascorbic acid (dissolved in physiological saline, w/v: 1 g/100 mL), 10 µL of IS (penicillin), and organic reagent (375 µL of methanol for serum, containing 0.2% formic acid). Then, the samples were vortexed and centrifuged at 12 000 rpm/min for 15 min. The preparation of serum was described in ***Preparation of serum*.** Separation was achieved at 30 °C using a Waters ACQUITY UPLC HSS T3 C18 column (100 ×2.1 mm, 1.8 μm). The mobile phase consisted of water containing 10 mM [ammonium](javascript:;) [acetate](javascript:;) (A) and methanol (B) at a flow rate of 0.25 mL**/**min. The gradient program used was as follows: 45% B from 0 to 0.5 min, 45%-62% B from 0.5 to 2 min, 62%-75% B from 2 to 15 min, 75%-90% B from 15 to 17 min, 90%-100% B from 17 to 18 min, and 100% B from 18 to 25 min. The injection volume was 3 μL. The optimized mass transition ion pairs (*m/z*), CE, and DE values for the BAs are shown in Table S7. The relevant parameters of the BA methodology in serum are described in Table S8.

**Lipid profiling analysis of serum and brain homogenate** Brain samples and serum samples were thawed on ice at 4 °C for 30-60 min. The excised brains (100 mg) were homogenized on ice. Then, homogenization buffer and an aliquot of 100 μL of serum were added to 600 μL of extraction solution (chloroform : methanol =3:1, V/V), and the mixture was ultrasonicated in an ice-water bath for 1 h, followed by the addition of 100 μL of water. The samples were then centrifuged at 12 000 rpm/min for 10 min at 4 °C, and the lipid phases (300 μL) were transferred into new tubes and evaporated to dryness. The residues were reconstituted in 400 μL of isopropyl alcohol/acetonitrile (1:1, V/V), followed by centrifugation. Then, the supernatant was transferred to an autosampler vial for further analysis.

An UltiMate^TM^ 3000 Rapid Separation LC (RSLC) system (Thermo Fisher Scientific, Waltham, MA) coupled *via* an electrospray ion source with a Q Exactive^TM^ system (Thermo Fisher Scientific, Waltham, MA) was used for lipid profiling. An aliquot of 1 μL of sample solution was injected into an ACQUITY UPLC HSS T3 C18 column (100 x 2.1 mm, 1.8 μm) maintained at 50 °C at a flow rate of 0.3 mL/min. The mobile phase consisted of solvent A (0.1% formic acid-water containing 2 mM ammonium formate) and solvent B (methanol) with a gradient elution (0-2 min, 20-30% B; 2-5 min, 30-45% B; 5-6.5 min, 45-55% B; 6.5-12 min, 55-65% B; 12-14 min, 65-85% B; 14-17.5 min, 85-100% B; and 17.5-18 min, 100-100% B). Global parameters were optimized across all analytes: The pos HESI-II spray voltages were 3.7 kV, the heated capillary temperature was 320 °C, the sheath gas pressure was 30 psi, the auxiliary gas setting was 10 psi, and the heated vaporizer temperature was 300 °C. The parameters of the full mass scan were as follows: resolution of 70 000, auto gain Contarget under 1×10^6^, maximum isolation time of 50 ms, and *m/z* range of 150-1500.

According to the retention time and exact mass, the raw LC-MS data were imported to Skyline software (<http://skyline.gs.washiington.edu>) for the relative quantification of the lipid species. The normalized data were exported into EZinfo 3.0 software for multivariate analysis. Orthogonal partial least squares discriminant analysis (OPLS-DA) and partial least-squares-discrimination analysis (PLS-DA) were used to visualize the differences for all groups and identify the pathologic biomarkers. The biomarkers were then filtered and confirmed by combining the results of the *VIP* values (*VIP*>1) and *t-*test (*p*<0.05).

**DNA extraction and V3 and V4 regions of 16S rRNA gene sequencing**

The total genomic DNA of faecal samples was extracted using the Mobio PowerSoil DNA Isolation Kit 100 (Qiagen, Germany) according to the manufacturer’s recommendations. The 16S rRNA genes were amplified using the specific primer for 16S V3-V4: 340F-805R to target the V3-V4 regions of 16S rRNA. PCR products were mixed in equimolar ratios and then purified with an Agencourt AMPure XP kit (Beckman Coulter, USA). The library quality was assessed on a QuantiFluor^TM^-ST fluorometer (Thermo Scientific, USA) and an Agilent Bioanalyzer 2100 system (Agilent Technologies, USA). The pooled library was sequenced on the Illumina MiSeq System with the sequencing strategy PE300 following the manufacturer’s instructions.

**Data processing and statistical analysis**

All values measured are presented as the means ± standard error of the mean. Comparisons between two groups were assessed using Student’s t-test. One-way ANOVA analysis followed by Dunnett or Tamhane's T2 test was employed to assess groups difference. Differences with *p*<0.05 were considered statistically significant. Statistical calculations were performed using IBM SPSS Statistics 22.

**References**

1. [Yang Y](https://www.ncbi.nlm.nih.gov/pubmed/?term=Yang%20Y%5BAuthor%5D&cauthor=true&cauthor_uid=24231264), [Wang HJ](https://www.ncbi.nlm.nih.gov/pubmed/?term=Wang%20HJ%5BAuthor%5D&cauthor=true&cauthor_uid=24231264), [Yang J](https://www.ncbi.nlm.nih.gov/pubmed/?term=Yang%20J%5BAuthor%5D&cauthor=true&cauthor_uid=24231264), [Brantner AH](https://www.ncbi.nlm.nih.gov/pubmed/?term=Brantner%20AH%5BAuthor%5D&cauthor=true&cauthor_uid=24231264), [Lower-Nedza AD](https://www.ncbi.nlm.nih.gov/pubmed/?term=Lower-Nedza%20AD%5BAuthor%5D&cauthor=true&cauthor_uid=24231264), [Si N](https://www.ncbi.nlm.nih.gov/pubmed/?term=Si%20N%5BAuthor%5D&cauthor=true&cauthor_uid=24231264), et al. Chemical profiling and quantification of Chinese medicinal formula Huang-Lian-Jie-Du decoction, a systematic quality control strategy using ultra high performance liquid chromatography combined with hybrid quadrupole-orbitrap and triple quadrupole mass spectrometers. J Chromatogr A. 2013;1321:88-99.

2. [Liu S](https://www.ncbi.nlm.nih.gov/pubmed/?term=Liu%20S%5BAuthor%5D&cauthor=true&cauthor_uid=24187871), [Wang N](https://www.ncbi.nlm.nih.gov/pubmed/?term=Wang%20N%5BAuthor%5D&cauthor=true&cauthor_uid=24187871), [Chen P](https://www.ncbi.nlm.nih.gov/pubmed/?term=Chen%20P%5BAuthor%5D&cauthor=true&cauthor_uid=24187871), [Li X](https://www.ncbi.nlm.nih.gov/pubmed/?term=Li%20X%5BAuthor%5D&cauthor=true&cauthor_uid=24187871), [Liu C](https://www.ncbi.nlm.nih.gov/pubmed/?term=Liu%20C%5BAuthor%5D&cauthor=true&cauthor_uid=24187871). Effect of Huanglianjiedu Tang on fever in rats induced by 2,4-dinitrophenol. J Tradit Chin Med, 2013;33:492-9.
