# Supplementary figures and images for: Huanglian Jiedu decoction remodels the periphery microenvironment to inhibit Alzheimer’s disease progression based on the “brain-gut” axis through multiple integrated omics
Source: Alzheimers Res Ther. 2021 Feb 12;13:44. doi: 10.1186/s13195-021-00779-7 (PMC7881564; doi:10.1186/s13195-021-00779-7)

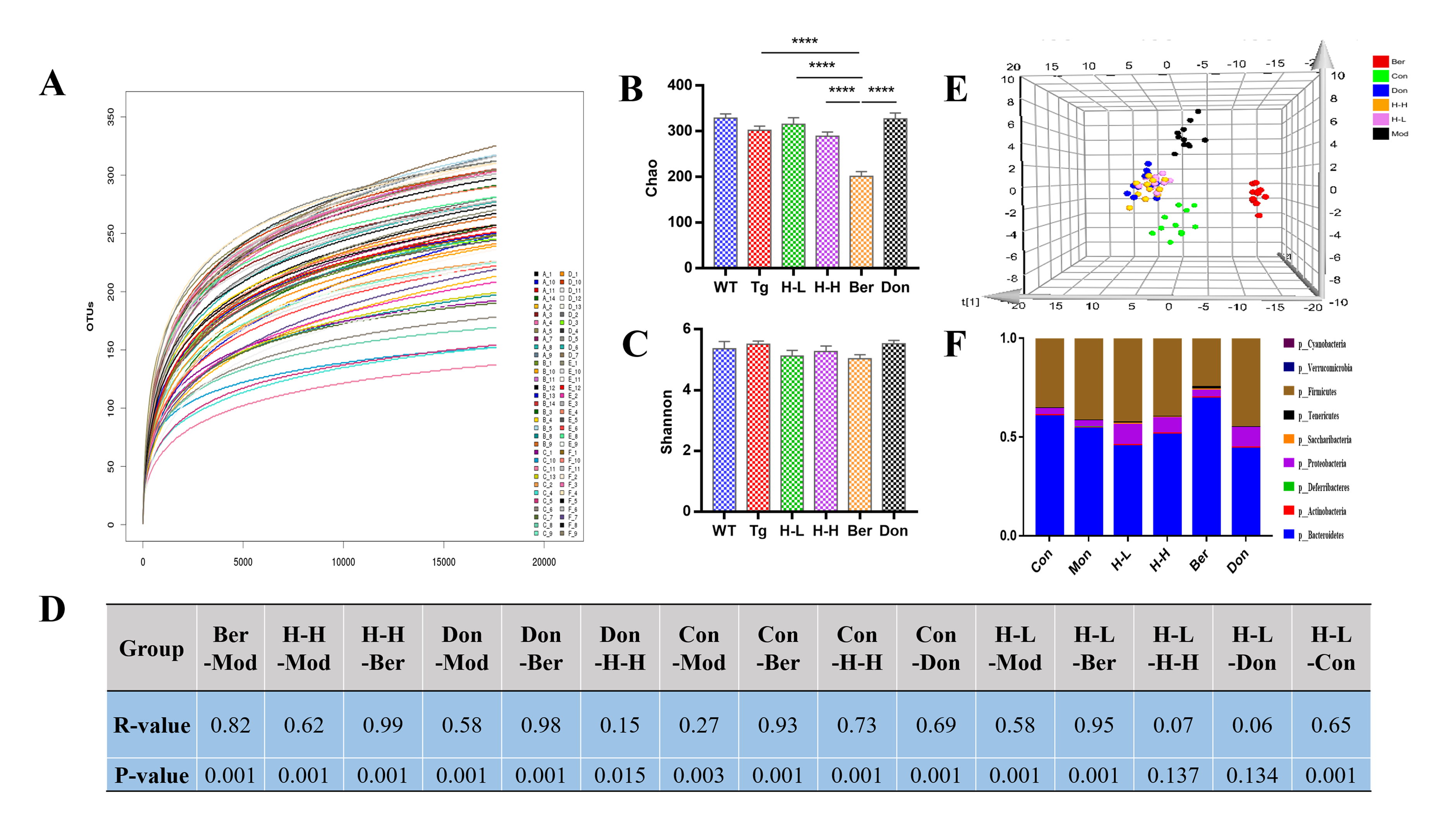

Supplement: Supplementary file 2 — Additional file 2: Table S1. Levels and ratios of Bile acids measured in the Tg mice (n = 5). Table S2. Peak areas of three main compounds in HLJDD. Table S3. The selected detecting ions, Declustering potential (DP), and collision energy (CE) of neurotransmitters. Table S4. The regression equations and linear range of NTs. Table S5. The selected detecting ions, declustering potential (DP), and collision energy (CE) of UFAs. Table S6. The regression equations and linear range of UFAs. Table S7. The selected detecting ions, declustering potential (DP), and collision energy (CE) of BAs. Table S8. The regression equations and linear range of BAs. Fig. S1. HLJDD altered the overall gut microbiota structure in Tg mice (n = 11). (a) Rarefaction analyses; (b) Chao1 index (data expressed as mean ± SEM); (c) Shannon index (data expressed as mean ± SEM); (d) ANOSIM analysis, R-value > 0 means the intra group difference was less than the inter group difference. (e) PLS-DA analyses; (f) Bacterial taxonomic profiling in the phylum level of gut microbiota. #P < 0.05 (comparing with the Mod group). Fig. S2. LEfSe rank plots of differentially abundant microbial clades in gut microbiome (n = 11). LDA scores (log 10) for differentially abundant microbial clades in stool among six groups and the threshold on the logarithmic LDA score for discriminative feature is > 3.0. Fig. S3. Venn diagram of the brain (a) and serum (b) showed the shared and unique correlated lipid compounds, in which each ellipse represented the potential lipid markers based on comparisons of the Tg group vs. drug treatment groups. Fig. S4. Profile of HPLC-UV chromatograms of mixed standard (a), HLJDD-1st (b) and HLJDD-7th (c) . peaks: 1. Geniposide; 2. Beiberine; 3. Baicalin. The chemical pattern of HLJDD aqueous did not change significantly after 7 days of preparation. [file 13195_2021_779_MOESM2_ESM.zip › Fig S1.png]

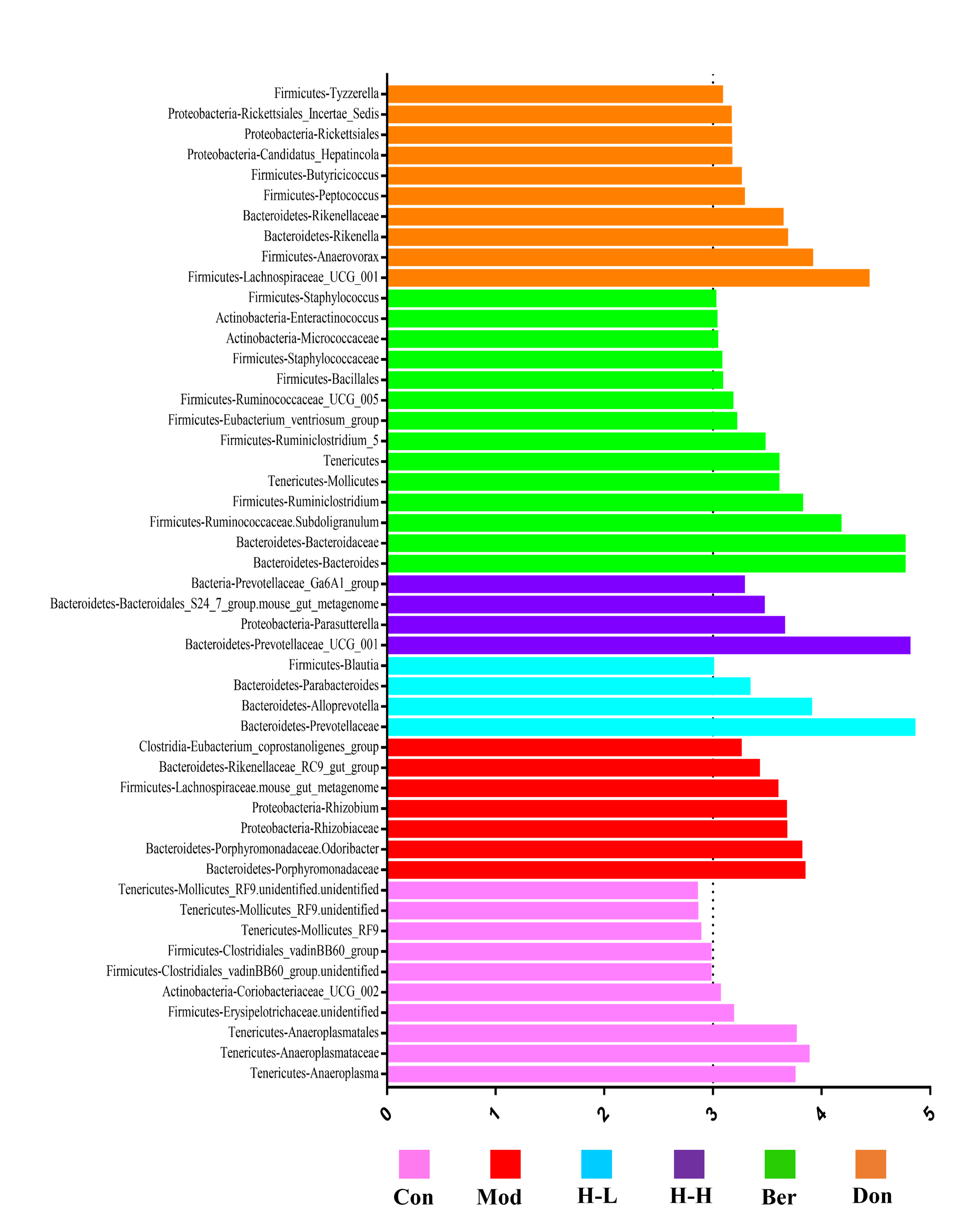

Supplement: Supplementary file 2 — Additional file 2: Table S1. Levels and ratios of Bile acids measured in the Tg mice (n = 5). Table S2. Peak areas of three main compounds in HLJDD. Table S3. The selected detecting ions, Declustering potential (DP), and collision energy (CE) of neurotransmitters. Table S4. The regression equations and linear range of NTs. Table S5. The selected detecting ions, declustering potential (DP), and collision energy (CE) of UFAs. Table S6. The regression equations and linear range of UFAs. Table S7. The selected detecting ions, declustering potential (DP), and collision energy (CE) of BAs. Table S8. The regression equations and linear range of BAs. Fig. S1. HLJDD altered the overall gut microbiota structure in Tg mice (n = 11). (a) Rarefaction analyses; (b) Chao1 index (data expressed as mean ± SEM); (c) Shannon index (data expressed as mean ± SEM); (d) ANOSIM analysis, R-value > 0 means the intra group difference was less than the inter group difference. (e) PLS-DA analyses; (f) Bacterial taxonomic profiling in the phylum level of gut microbiota. #P < 0.05 (comparing with the Mod group). Fig. S2. LEfSe rank plots of differentially abundant microbial clades in gut microbiome (n = 11). LDA scores (log 10) for differentially abundant microbial clades in stool among six groups and the threshold on the logarithmic LDA score for discriminative feature is > 3.0. Fig. S3. Venn diagram of the brain (a) and serum (b) showed the shared and unique correlated lipid compounds, in which each ellipse represented the potential lipid markers based on comparisons of the Tg group vs. drug treatment groups. Fig. S4. Profile of HPLC-UV chromatograms of mixed standard (a), HLJDD-1st (b) and HLJDD-7th (c) . peaks: 1. Geniposide; 2. Beiberine; 3. Baicalin. The chemical pattern of HLJDD aqueous did not change significantly after 7 days of preparation. [file 13195_2021_779_MOESM2_ESM.zip › Figure S2.png]

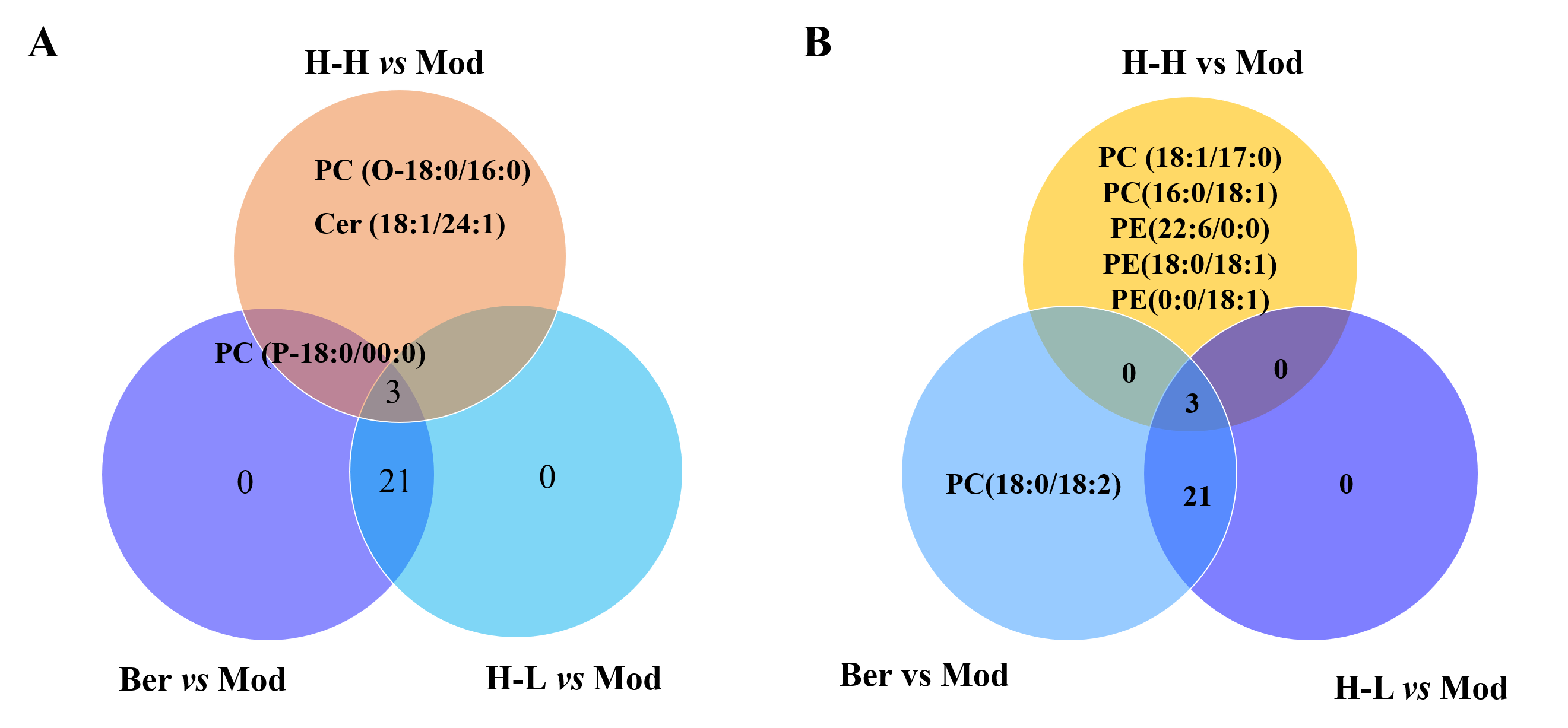

Supplement: Supplementary file 2 — Additional file 2: Table S1. Levels and ratios of Bile acids measured in the Tg mice (n = 5). Table S2. Peak areas of three main compounds in HLJDD. Table S3. The selected detecting ions, Declustering potential (DP), and collision energy (CE) of neurotransmitters. Table S4. The regression equations and linear range of NTs. Table S5. The selected detecting ions, declustering potential (DP), and collision energy (CE) of UFAs. Table S6. The regression equations and linear range of UFAs. Table S7. The selected detecting ions, declustering potential (DP), and collision energy (CE) of BAs. Table S8. The regression equations and linear range of BAs. Fig. S1. HLJDD altered the overall gut microbiota structure in Tg mice (n = 11). (a) Rarefaction analyses; (b) Chao1 index (data expressed as mean ± SEM); (c) Shannon index (data expressed as mean ± SEM); (d) ANOSIM analysis, R-value > 0 means the intra group difference was less than the inter group difference. (e) PLS-DA analyses; (f) Bacterial taxonomic profiling in the phylum level of gut microbiota. #P < 0.05 (comparing with the Mod group). Fig. S2. LEfSe rank plots of differentially abundant microbial clades in gut microbiome (n = 11). LDA scores (log 10) for differentially abundant microbial clades in stool among six groups and the threshold on the logarithmic LDA score for discriminative feature is > 3.0. Fig. S3. Venn diagram of the brain (a) and serum (b) showed the shared and unique correlated lipid compounds, in which each ellipse represented the potential lipid markers based on comparisons of the Tg group vs. drug treatment groups. Fig. S4. Profile of HPLC-UV chromatograms of mixed standard (a), HLJDD-1st (b) and HLJDD-7th (c) . peaks: 1. Geniposide; 2. Beiberine; 3. Baicalin. The chemical pattern of HLJDD aqueous did not change significantly after 7 days of preparation. [file 13195_2021_779_MOESM2_ESM.zip › Figure S3.png]

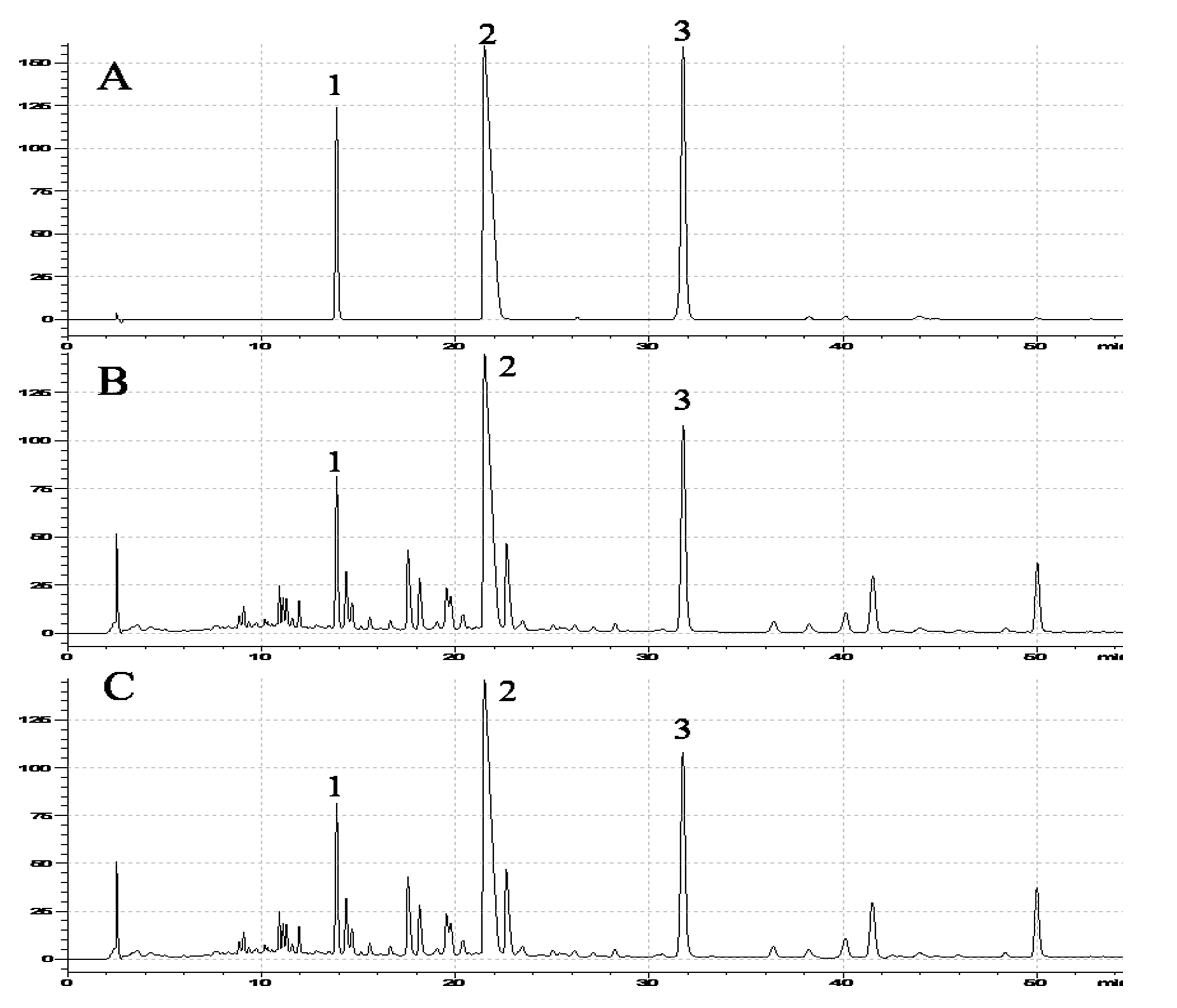

Supplement: Supplementary file 2 — Additional file 2: Table S1. Levels and ratios of Bile acids measured in the Tg mice (n = 5). Table S2. Peak areas of three main compounds in HLJDD. Table S3. The selected detecting ions, Declustering potential (DP), and collision energy (CE) of neurotransmitters. Table S4. The regression equations and linear range of NTs. Table S5. The selected detecting ions, declustering potential (DP), and collision energy (CE) of UFAs. Table S6. The regression equations and linear range of UFAs. Table S7. The selected detecting ions, declustering potential (DP), and collision energy (CE) of BAs. Table S8. The regression equations and linear range of BAs. Fig. S1. HLJDD altered the overall gut microbiota structure in Tg mice (n = 11). (a) Rarefaction analyses; (b) Chao1 index (data expressed as mean ± SEM); (c) Shannon index (data expressed as mean ± SEM); (d) ANOSIM analysis, R-value > 0 means the intra group difference was less than the inter group difference. (e) PLS-DA analyses; (f) Bacterial taxonomic profiling in the phylum level of gut microbiota. #P < 0.05 (comparing with the Mod group). Fig. S2. LEfSe rank plots of differentially abundant microbial clades in gut microbiome (n = 11). LDA scores (log 10) for differentially abundant microbial clades in stool among six groups and the threshold on the logarithmic LDA score for discriminative feature is > 3.0. Fig. S3. Venn diagram of the brain (a) and serum (b) showed the shared and unique correlated lipid compounds, in which each ellipse represented the potential lipid markers based on comparisons of the Tg group vs. drug treatment groups. Fig. S4. Profile of HPLC-UV chromatograms of mixed standard (a), HLJDD-1st (b) and HLJDD-7th (c) . peaks: 1. Geniposide; 2. Beiberine; 3. Baicalin. The chemical pattern of HLJDD aqueous did not change significantly after 7 days of preparation. [file 13195_2021_779_MOESM2_ESM.zip › Figure S4.png]
